# Physical activity level and stroke risk in US population: A matched case–control study of 102,578 individuals

**DOI:** 10.1002/acn3.51511

**Published:** 2022-01-30

**Authors:** Sherief Ghozy, Ahmad Helmy Zayan, Amr Ehab El‐Qushayri, Kate Elizabeth Parker, Joseph Varney, Kevin M. Kallmes, Sara Morsy, Alzhraa Salah Abbas, Jose Danilo B. Diestro, Adam A. Dmytriw, Jaffer Shah, Ameer E. Hassan, Sheikh Mohammed Shariful Islam

**Affiliations:** ^1^ Department of Neuroradiology Mayo Clinic Rochester Minnesota USA; ^2^ Nuffield Department of Primary Care Health Sciences and Department for Continuing Education (EBHC program) Oxford University Oxford UK; ^3^ Faculty of Medicine Menoufia University Menoufia Egypt; ^4^ Faculty of Medicine Minia University Minia Egypt; ^5^ Institute for Physical Activity and Nutrition (IPAN), School of Exercise and Nutrition Sciences Deakin University Melbourne Vic. Australia; ^6^ School of Medicine American University of the Caribbean Cupecoy Sint Maarten; ^7^ Nested Knowledge St. Paul Minnesota USA; ^8^ Superior Medical Experts St. Paul Minnesota USA; ^9^ Medical Biochemistry and Molecular Biology Department, Faculty of Medicine Tanta University Tanta Egypt; ^10^ School of Chemistry and Biosciences, Faculty of Life Sciences University of Bradford Bradford UK; ^11^ Radiology Department, Central Hospital of University of Montreal University of Montreal Montreal QC Canada; ^12^ Neuroendovascular Program Massachusetts General Hospital, Harvard Medical School Boston Massachusetts USA; ^13^ Medical Research Center Kateb University Kabul Afghanistan; ^14^ Department of Neurology University of Texas Rio Grande Valley Harlingen Texas USA

## Abstract

**Background:**

Stroke has been linked to a lack of physical activity; however, the extent of the association between inactive lifestyles and stroke risk has yet to be characterized across large populations.

**Purpose:**

This study aimed to explore the association between activity‐related behaviors and stroke incidence.

**Methods:**

Data from 1999 to 2018 waves of the concurrent cross‐sectional National Health and Nutrition Examination Survey (NHANES) were extracted. We analyzed participants characteristics and outcomes for all participants with data on whether they had a stroke or not and assessed how different forms of physical activity affect the incidence of disease.

**Results:**

Of the 102,578 individuals included, 3851 had a history of stroke. A range of activity‐related behaviors was protective against stroke, including engaging in moderate‐intensity work over the last 30 days (OR = 0.8, 95% CI = 0.7–0.9; *P* = 0.001) and vigorous‐intensity work activities over the last 30 days (OR = 0.6, 95% CI = 0.5–0.8; *P* < 0.001), and muscle‐strengthening exercises (OR = 0.6, 95% CI = 0.5–0.8; *P* < 0.001). Conversely, more than 4 h of daily TV, video, or computer use was positively associated with the likelihood of stroke (OR = 11.7, 95% CI = 2.1–219.2; *P* = 0.022).

**Conclusion:**

Different types, frequencies, and intensities of physical activity were associated with reduced stroke incidence, implying that there is an option for everyone. Daily or every other day activities are more critical in reducing stroke than reducing sedentary behavior duration.

## Introduction

In 2019, there were 101 million stroke cases, 143 million stroke‐related disability‐adjusted life years, and 6.55 million deaths.^1^ Each year, approximately 795,000 Americans experience a stroke, with roughly 185,000 of those individuals succumbing to the illness.^2^ Because of the high incidence of stroke, one in every six deaths from cardiovascular disease are stroke‐related.^3^ There are many challenges for stroke treatment specifically in low‐ and middle‐income countries mainly due to high cost and unavailable infrastructure.^4^ That is why, prevention is considered more effective than treatment for stroke.^5^ One of the most reported method of prevention is through physical activity.^5^

Physical activity (and exercise) has long been recognized as a potential behavioral approach for preventing stroke. Review highlights that physical activity and exercise provide strong preventative effects on stroke recurrence.^6^ The American Stroke Association (ASA) noted that physical activity “reduces blood pressure, improves endothelial function, reduces insulin resistance, improves lipid metabolism, and may help reduce weight”.^7^ Some large‐scale observational studies have demonstrated a dose–response risk reduction of stroke with intensity and duration of physical activity^8^, ^9^ or among the fit and highly active individuals compared to unfit or low‐active individuals.^10^ However, no studies have looked at the direct association between a broad range of activity‐related behaviors and stroke risk.

It is well understood that individuals are unique in their activity‐related behavior habits and preferences. Thus, it is crucial to identify the direct association between various activity‐related behaviors with the likelihood of stroke. Furthermore, the dose–response relationship between various volumes, intensities, and domains of activity‐related behaviors on reducing stroke is poorly understood.^11^ Here, we present the first nationwide health survey analysis of a range of activity‐related behaviors and their impact on association with stroke risk. The findings from this study will contribute toward establishing the most effective dose of a broad range of activity‐related behaviors on stroke risk among the adult population and serve as a basis for experimental research to determine causative effects of increasing physical activity and reducing sedentary behavior on reduction in stroke risk.

## Methods

### Data source

A cross‐sectional study was performed using the National Health and Nutrition Examination Survey (NHANES) developed by the Centers for Disease Control and Prevention (CDC) in the USA. Nearly 10,000 participants completed a self‐report survey every 2 years since 1999.^12^ We combined responses from 1999 to 2006, 2007 to 2018, and 2011 to 2016 for analysis.^12^

### Selection of cases and controls

We selected only participants diagnosed with stroke; stroke was self‐reported as it was assessed through medical conditions questionnaire. We included all individuals who answered either “yes” or “no” to the following question: “Has a doctor or other health professional ever told you that you had a stroke?” We excluded participants who answered this question with any other response, such as “do not know,” refused to answer the question or participants with missing data. We defined cases as participants who answered yes to the question while controls are those who answered no.

Based on these criteria, 102,578 individuals were eligible for study inclusion; out of them, 3851 had a stroke history, and 98,727 individuals did not. Questions assessing physical activity have changed over different years, and as such, all years using the same questionnaire were grouped during analysis. Accordingly, three intervals were present: 1999–2006, 2007–2018, and 2011–2016 (Table S1). Following the propensity score matching, 7702 individuals were included in the logistic regression model.

### Variables included in the analysis

Population characteristics were extracted from the demographic data of survey participants and outcome variables were extracted from survey responses. We included participants from 10 surveys conducted between 1999 and 2018. The variables collected were: patient characteristics: age, gender, race, educational, marital status, overweight status; history of congestive heart failure, coronary heart disease, angina, and heart attack.

*Physical activity variables:* 1‐Walked or bicycled over past 30 days, duration of walking or bicycling per day, and number of times walked or bicycled. 2‐Tasks around home yard past 30 days, duration of tasks around home yard in past 30 days and number of times performed tasks around home yard in past 30 days. 3‐Vigorous activity over past 30 days, days of vigorous activity, and minutes of vigorous activity. 4‐Vigorous recreational activity over past 30 days, days of vigorous recreational activity, and minutes of vigorous recreational activity. 5‐Moderate activity over past 30 days, days of moderate activity, and minutes of moderate activity. 6‐Moderate recreational activity over past 30 days, days of moderate recreational activity, and minutes of moderate recreational activity. 7‐Average level of physical activity each day. 8‐Muscle strengthening activities and number of times muscle strengthening activities in past 30 days' activity. 9‐Comparison of activity last month or last year, compare activity with others in the same age, compare activity with 10 years ago. 10‐Minutes sedentary activity. 11‐Hours watch TV or videos past 30 days. 12‐Hours use computer past 30 days. The survey tool description and response options are summarized in Table S2.

### Statistical analysis

All data were analyzed using R software version 4.0.2 using the packages “Rcmdr”, “glm2”, and “MatchIt”.^13^ According to the stroke incidence status, all variables were represented as frequencies and percentages, with chi‐squared tests used for testing the difference. To reduce confounding bias due to variables between case (stroke patients) and comparison cohort (non‐stroke controls), we used the propensity score matching method for all baseline characteristics (age, gender, race, education, marital status, history of cardiovascular conditions) to select subjects without stroke history on 1:1 ratio for each stroke patient. Logistic regression was used to identify any possible association between activity‐related behavior participation and stroke incidence among the matched sample.^14^ Regression results were expressed as odds ratios (ORs) and 95% confidence interval (95% CI). *P*‐value <0.05 was considered significant for all statistical tests.

## Results

Of the 102,578 individuals included, 3851 had a history of stroke, and 98,727 were controls. Stroke patients were significantly older compared to controls, and more than two‐thirds were aged ≥60 years. Moreover, a substantially higher proportion of stroke patients were males during 1999–2006 and 2007–2018 surveys. Non‐Hispanic white was the most presented race in stroke patients. In addition, most participants were married and had an education level of 12th grade (without a diploma) or less. Stroke patients had a significantly higher prevalence of comorbidities, including overweight, congestive heart failure, coronary heart disease, angina, and heart attack, when compared with controls (Table S1).

### Light‐ and moderate‐intensity aerobic activities

As shown in Table S3, there were significant differences in the performance and frequency of all assessed activities among the stroke and non‐stroke groups, except for matched individuals' work‐related moderate‐intensity activities and minutes of walking or bicycling for transportation.

**Figure 1:**
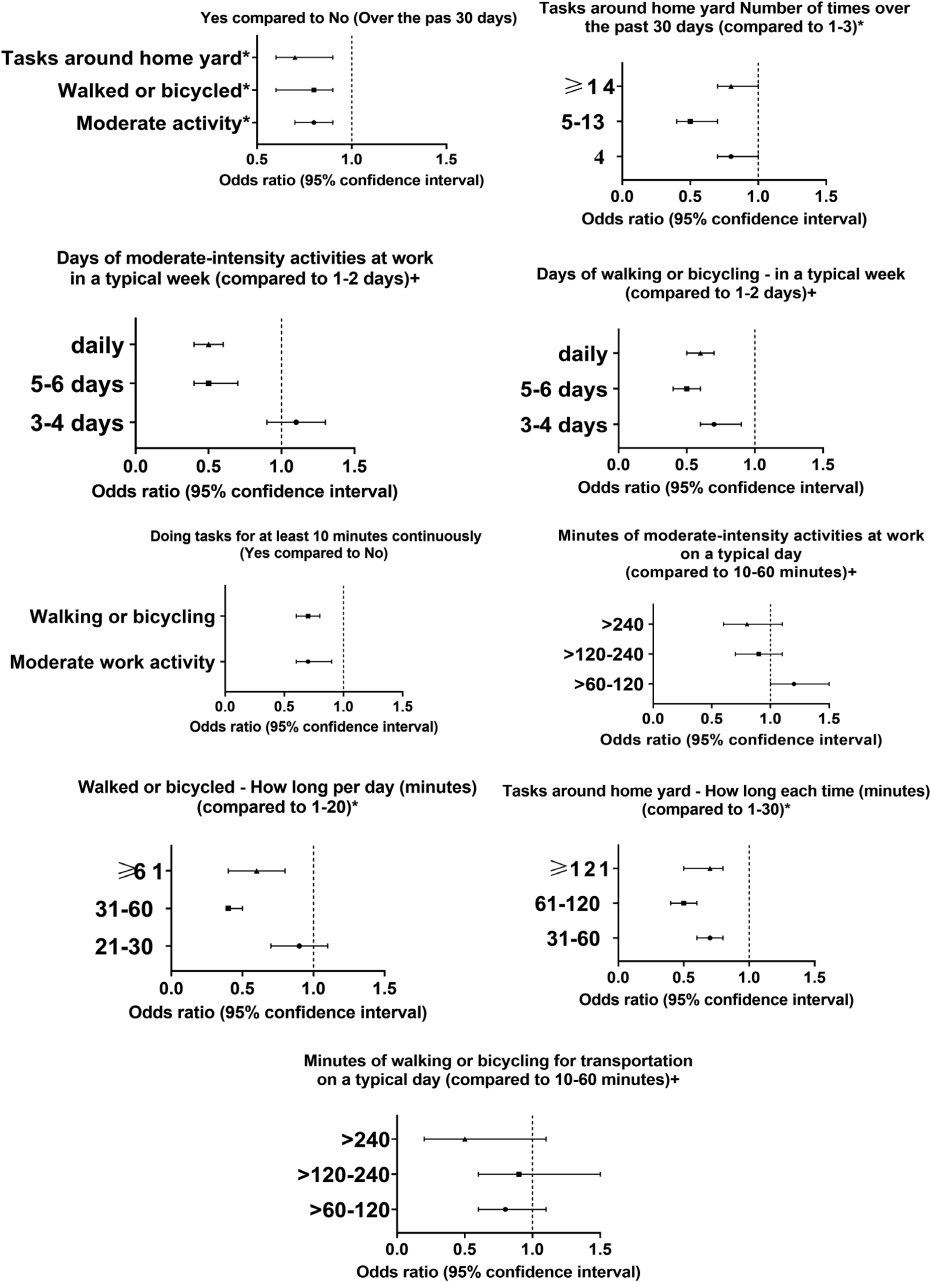
Association between light‐ to moderate‐intensity activities and stroke odds

Figure 1 shows the results of the association between aerobic activities and stroke odds in the matched sample. In general, participants who performed moderate‐intensity work activities over the past 30 days had a lower odds of developing stroke (OR = 0.8, 95% CI = 0.7–0.9; *P* = 0.001). Moreover, walking or bicycling over the past 30 days was associated with reduced odds of stroke (OR = 0.8, 95% CI = 0.6–0.9; *P*‐ = 0.003), with the largest reduction in odds observed when the duration of bicycling or walking was between 31 and 60 min daily (OR = 0.4, 95% CI = 0.4–0.5; *P* < 0.001). Furthermore, performing tasks around the home or yard over the past 30 days was associated with a significantly lower odds of stroke (OR = 0.7, 95% CI = 0.6–0.9; *P* < 0.001). This would indicate that moderate‐intensity aerobic activities can reduce the likelihood of stroke by 20%, and this can be as high as 60% with a daily activity for 31–60 min.

In terms of the activity duration/frequency, performing moderate‐intensity activities at work, for at least 10 min continuously, was associated with decreased stroke odds (OR = 0.7, 95% CI = 0.6–0.9; *P* < 0.001). In a typical week, individuals who performed daily (OR = 0.6, 95% CI = 0.4–0.7; *P* < 0.001) or 5–6 days (OR = 0.6, 95% CI = 0.4–0.7; *P* < 0.001) of moderate‐intensity activities at work had lower rates of stroke. However, there was no significant reduction in stroke rates in terms of the duration of moderate‐intensity activities at work on a typical day. Additionally, walking or bicycling, for at least 10 minutes continuously, was associated with a decreased odds of stroke (OR = 0.7, 95% CI = 0.6–0.9; *P* < 0.001) and the significant reduction in odds was observed when walked or bicycled for 5–6 days per week (OR = 0.5, 95% CI = 0.4–0.7; *P* < 0.001) or daily (OR = 0.5, 95% CI = 0.4–0.6; *P* < 0.001). However, no significant difference was found when comparing different durations (min) of walking or bicycling on a typical day. Akin to that, performing tasks around home with a duration of 61–120 min per task or doing those tasks 5–13 times over the past 30 days was associated with the largest reduction in odds of stroke (*P* < 0.001) (Table S4).

### Vigorous‐intensity aerobic activities

There were significant differences in the performance and frequency of all assessed vigorous‐intensity activities among the stroke group and non‐stroke group, except for matched individuals' vigorous‐intensity activities at work for at least 10 min continuously (Table S3).

**Figure 2:**
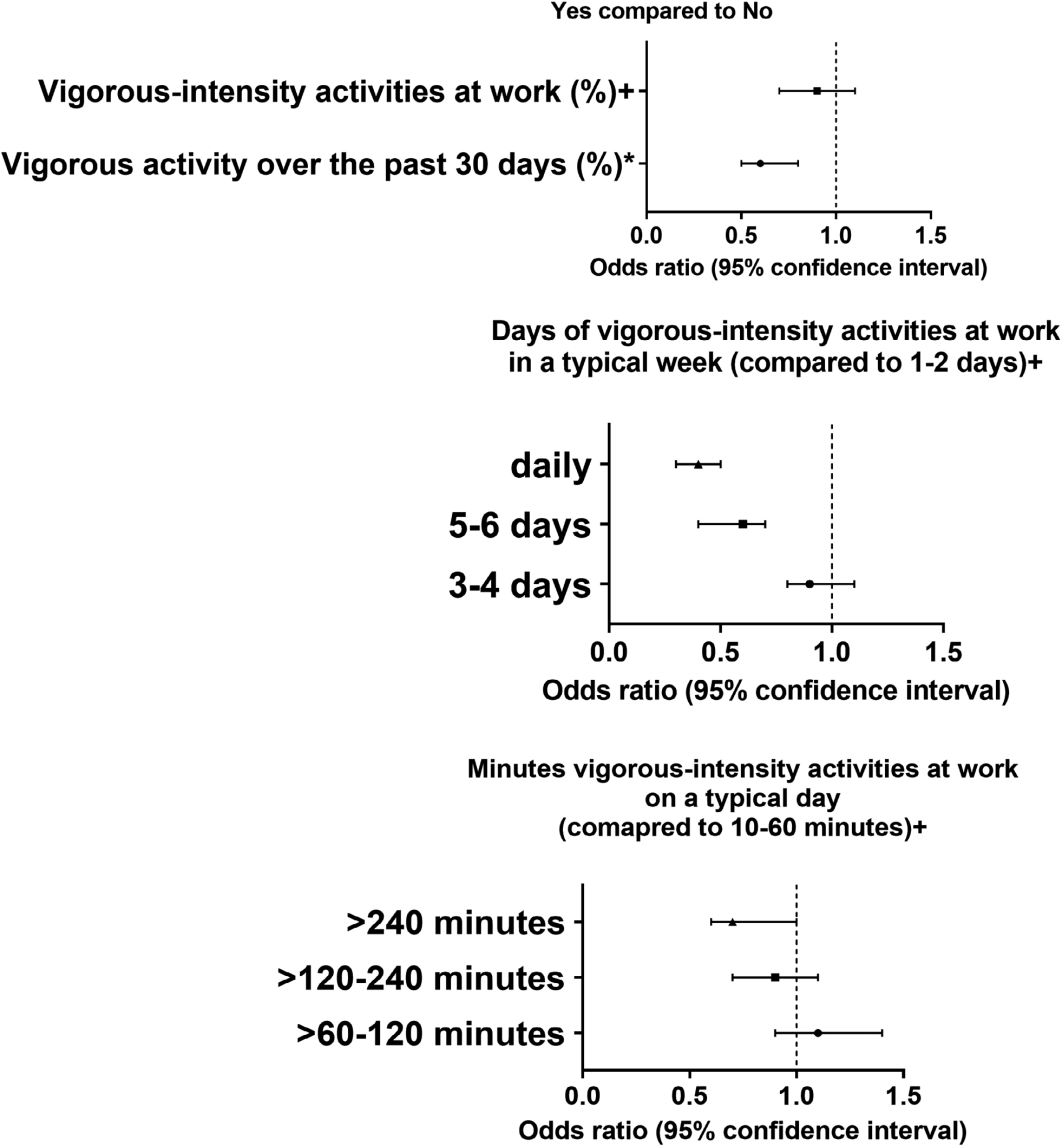
Association between vigorous‐intensity activities and stroke odds

Individuals who performed vigorous work activities over the past 30 days had a lower odds of stroke (OR = 0.6, 95% CI = 0.5–0.8; *P* < 0.001) compared to those who did not. Moreover, performing vigorous activities at work for more than 240 min in a typical day (OR = 0.7, 95% CI = 0.6–1.0; *P* = 0.037), daily in a typical week (OR = 0.4, 95% CI = 0.3–0.5; *P* < 0.001), and for 5–6 days in a typical week (OR = 0.6, 95% CI = 0.4–0.7; *P* < 0.001) were all associated with a lower odds of stroke compared to 10–60 minutes in a typical day, and 1–2 days in a typical week, respectively. However, vigorous‐intensity activities at work, for at least 10 min continuously, were not associated with a reduction in stroke rates (OR = 0.9, 95% CI = 0.7–1.1; *P* = 0.328) (Figure 2).

### Recreational activities

There were significant differences in the performance and frequency of all assessed recreational activities among the stroke group and non‐stroke group, whether in matched or non‐matched groups (Table S5).

**Figure 3:**
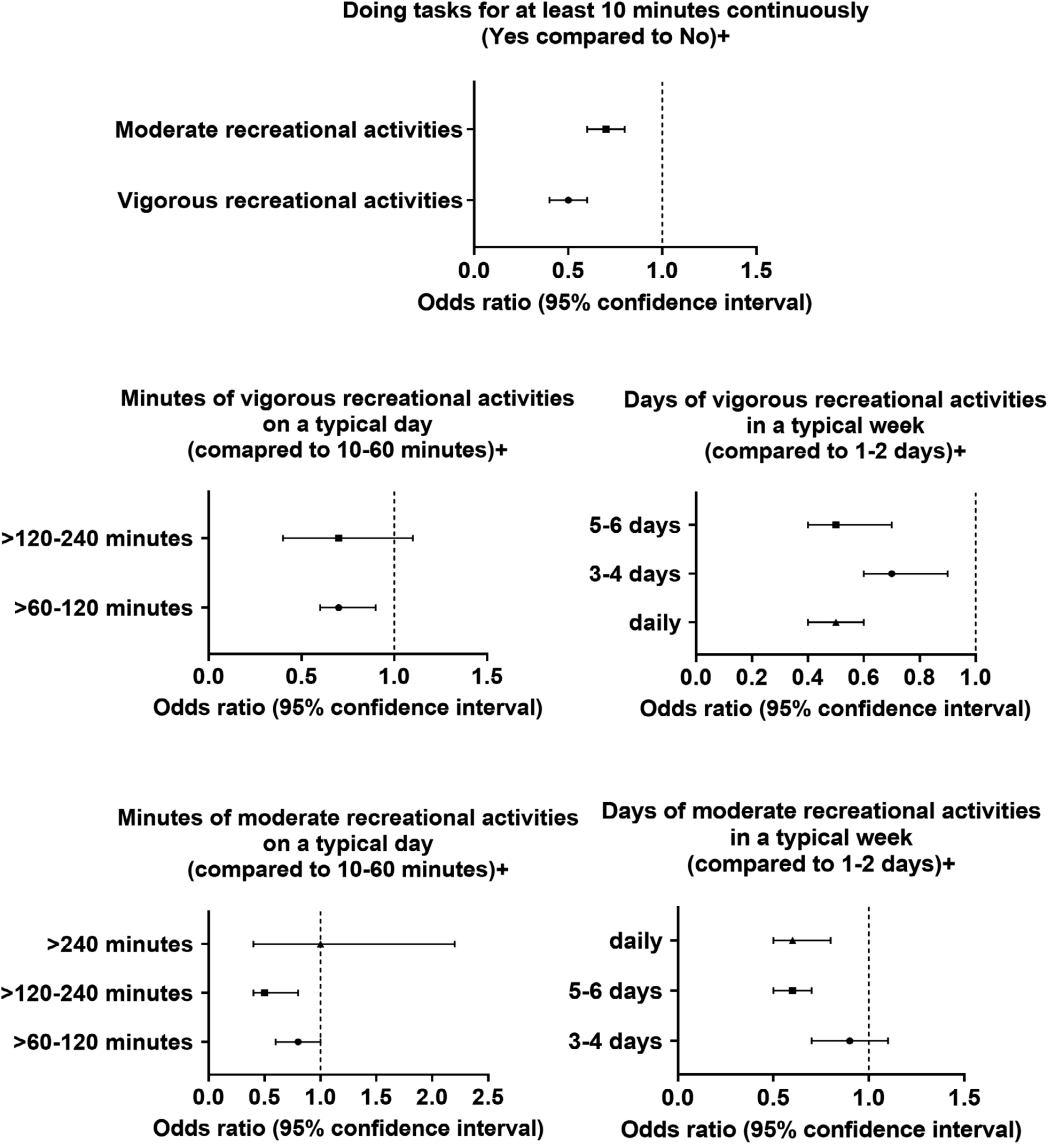
Association between recreational activities and stroke odds

Vigorous (OR = 0.5, 95% CI = 0.4–0.6; *P* < 0.001) and moderate (OR = 0.7, 95% CI = 0.6–0.8; *P* < 0.001) recreational activities, for at least 10 minutes continuously, were associated with lower stroke rates (Table S6). In a typical week, all frequencies of vigorous recreational activities beyond 2 days (as compared to 1–2 days) were associated with a significant reduction in stroke incidence, whether 3–4 days (OR = 0.7, 95% CI = 0.6–0.9; *P* = 0.001), 5–6 days (OR = 0.5, 95% CI = 0.4–0.7; *P* < 0.001), or every day (OR = 0.5, 95% CI = 0.4–0.6; *P* < 0.001). When it comes to the duration of vigorous recreational activities on a typical day, >60 to 120 min was found to be associated with the largest reduction in odds of stroke (OR = 0.7, 95% CI = 0.6–0.9; *P* = 0.018), while durations above this interval did not show similar effect. For the moderate recreational activities, only 5–6 times per week (OR = 0.6, 95% CI = 0.5–0.7; *P* < 0.001) and daily (OR = 0.6, 95% CI = 0.5–0.8; *P* < 0.001) frequencies were associated with a reduction in stroke odds. When it comes to the duration of moderate recreational activities on a typical day, >60 to 120 min (OR = 0.6, 95% CI = 0.5–0.7; *P* < 0.001) and >120 to 240 min (OR = 0.6, 95% CI = 0.5–0.7; *P* < 0.001) were associated with the greatest reduction in odds of stroke (as compared to 10–60 min), while durations above this interval did not show a similar association (Figure 3).

### Muscle‐strengthening activities

**Figure 4:**
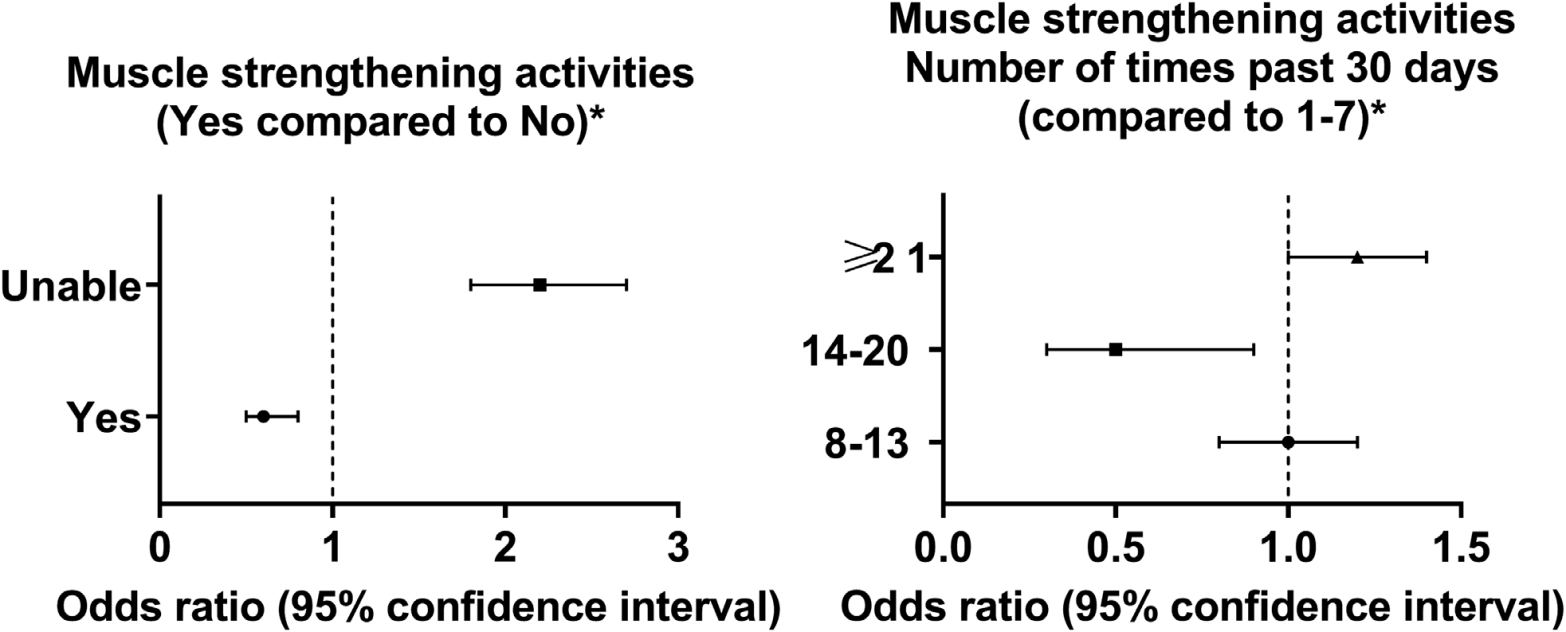
Association between muscle strengthening activities and stroke odds

There were significant differences in the performance and frequency of muscle‐strengthening activities among the stroke and non‐stroke groups (Table S7). Participants who performed muscle‐strengthening activities had lower odds of stroke (OR = 0.6, 95% CI = 0.5–0.8; *P* < 0.001). Doing muscle‐strengthening activities 14–20 times over the past 30 days was associated with a significant lower odds of stroke (OR = 0.5, 95% CI = 0.3–0.9; *P* = 0.012); however, a higher frequency of muscle‐strengthening activities did not show a similar benefit (OR = 1.2, 95% CI = 1.0–1.4; *P* = 0.102) (Figure 4; Table S8).

### Sedentary behavior

**Figure 5:**
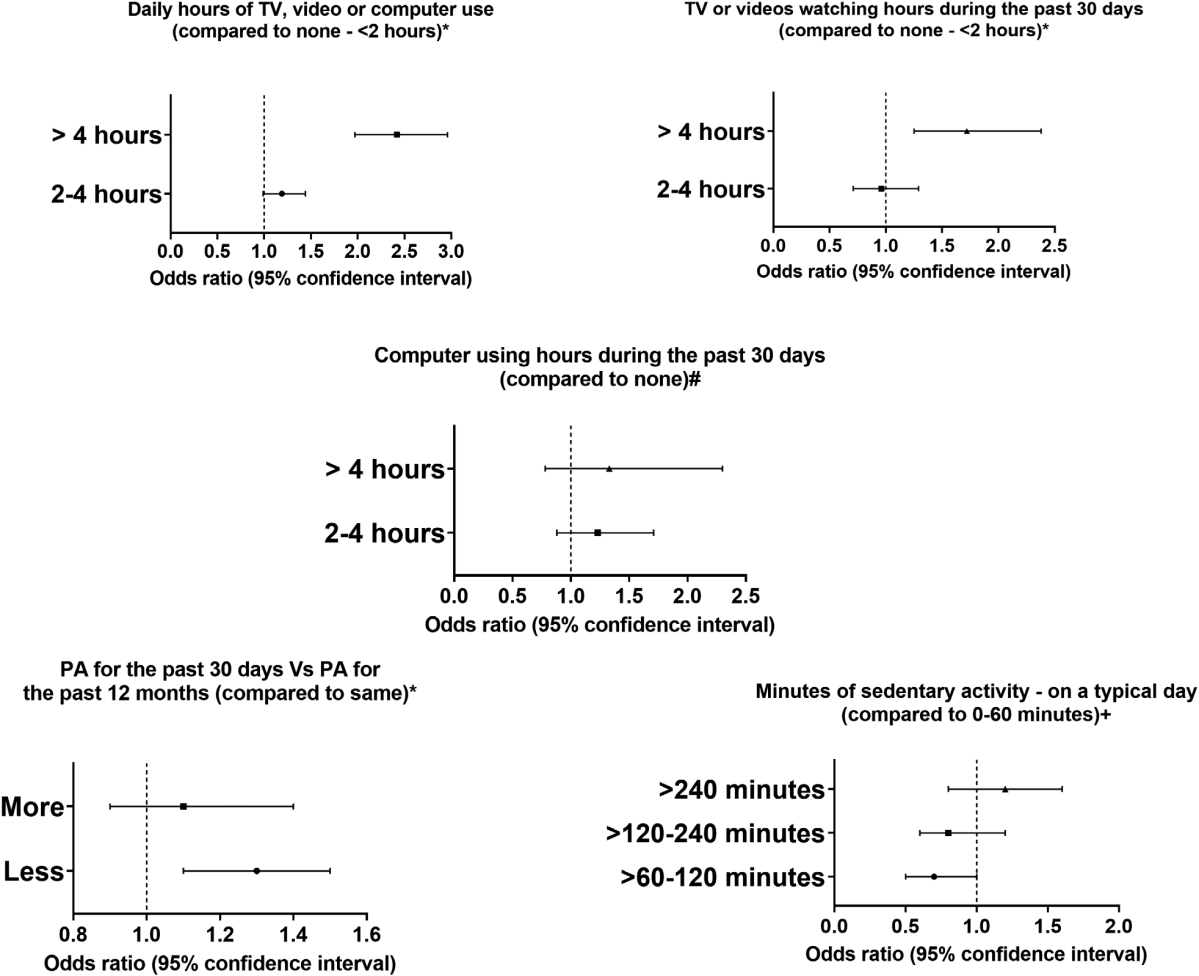
Association between sedentary behavior and stroke odds

There were significant differences in the performance and frequency of all assessed recreational sedentary behaviors among the stroke group and non‐stroke group (Table S9). Daily TV, video, or computer use for more than 4 h was associated with a considerable increase in stroke odds (OR = 2.42, 95% CI = 1.79–2.96; *P* < 0.001), while the effect of lesser durations did not reach a statistically significant level. Conversely, the minutes of sedentary activities on a typical day and the hours of computer using; were not significant predictors of stroke. Compared to “none to less than two hours”, watching TV, or videos in the past 30 days for more than 4 h was associated with higher odds of stroke (OR = 1.72, 95% CI = 1.25–2.38; *P* < 0.001), while less durations were not associated with stroke incidence (Figure 5). This would suggest that the combined use of TV, video, or computer becomes harmful when used daily for durations of more than 4 h (Table S10).

### Activity levels' comparisons

**Figure 6:**
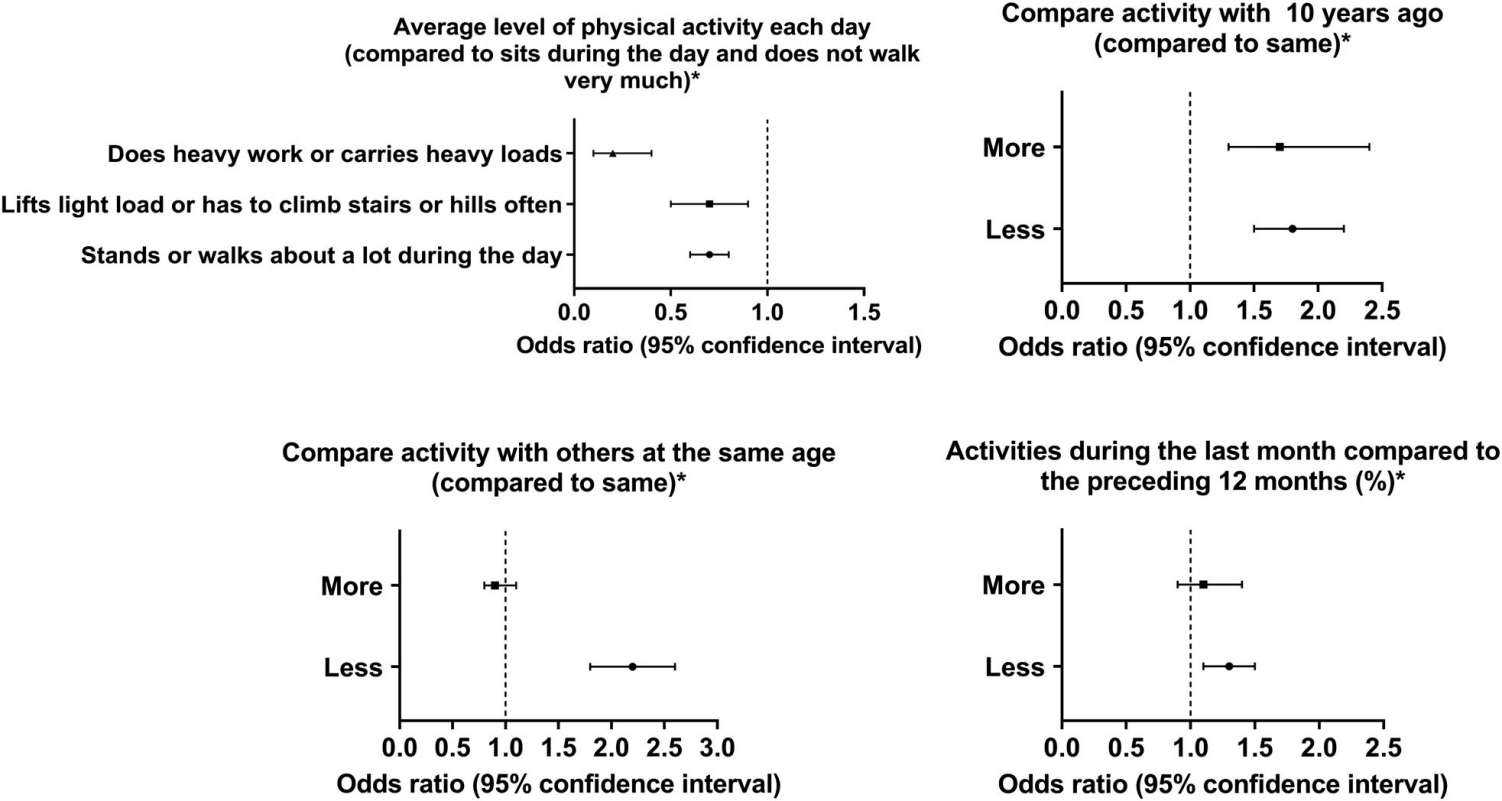
Association between activity levels' changes and stroke odds

There were significant differences in the activity levels among the stroke group and non‐stroke group, whether in matched or non‐matched samples (Table S11). In terms of average levels of physical activities, individuals doing heavy work or carrying heavy loads were associated with the lowest odds of stroke prevalence (OR = 0.2, 95% CI = 0.1–0.4; *P* < 0.001) as compared to those sitting during the day with “not walking very much”. Additionally, doing less activities during the last month compared to the preceding 12 months (OR = 1.3, 95% CI = 1.1–1.5; *P* = 0.002), less activity compared to 10 years ago (OR = 1.8, 95% CI = 1.5–2.2; *P* < 0.001), and less activity levels compared to others at the same age (OR = 2.2, 95% CI = 1.8–2.6; *P* < 0.001); were all associated with a significant increase in stroke odds. Doing more activities compared to 10 years ago also increased stroke odds (OR = 1.7, 95% CI = 1.3–2.4; *P* < 0.001) (Figure 6). This means that maintaining the activity level with no reduction is much more important than increasing it, and the activity should be tailored according to each life stage (Table S12).

## Discussion

Our findings showed that 30–60 min of daily walking or bicycling, performing tasks around the home yard for 60–120 min per task, or 5–13 times over the past 30 days, moderate work activities over the past 30 days were all associated with a reduced likelihood of stroke, compared to not engaging in those behaviors. In terms of work intensity, moderate work activities were associated with a significant reduction in stroke odds, whereas vigorous work activity contradicted results based on the duration or frequency. Vigorous work activities can reduce stroke odds by 40%, and the only way to get higher up to 60% is to do it daily, which may not be the best option for advanced age groups or those with specific co‐morbidities. Muscle‐strengthening activities can reduce the likelihood of stroke by 40%. The optimum frequency would be 3–5 days per week (14–20 per month) to enhance this reduction up to 50%, while fewer or more times did not show a similar benefit. Compared to the past or the age‐matched individuals, a reduction in physical activity was a significant predictor of stroke incidence, while its increase did not show a significant benefit. Regular walking or using a bicycle to get to and from places is an easy and effective preventive measure. The current findings support the importance of maintaining physical activity and avoiding sedentary behaviors and represent a call to action for providers to focus on lifestyle‐related strategies in populations at risk of stroke.

The INTERSTROKE study has identified the five key risk factors for stroke; among these, the single risk factor that accounts for more than 80% of the global stroke burden is physical inactivity.^15^ Nor is this problem retreating: as social media, electronic/remote school, and work, and video gaming become an everyday part of life, we need to understand its implications on physical activity. In one decade, the usage of electronics has increased threefold, and now is the activity in which individuals spend the second‐most time, after sleep.^16^ Thus, without intervention, providers should anticipate an ongoing decrease in physical activity, a rise in sedentary behavior, and an associated increase in stroke rates.

While many factors associated with an increased risk of stroke (non‐Hispanic White, uneducated, married, and those ≥60 years of age) are challenging to address, the most crucial recognizable risk factor for both ischemic and hemorrhagic strokes is hypertension.^17^ A previous study reported a linear relationship between the participant's blood pressure and stroke risk.^18^ Since reductions in participant's blood pressure are directly correlated to an increase in physical activity,^19^, ^20^, ^21^ the antihypertensive effects of physical activity could be a major contributor to reducing stroke occurrence. These effects include favorable lipid metabolism (increased HDL, lowered LDL), improved function of endothelial tissues, and decreased blood viscosity.^22^, ^23^, ^24^ Clotting factors that are also positively affected by physical activity (increased aggregation of platelets, increased fibrinolysis, increased plasma tissue plasminogen activator activity, and decreased fibrinogen levels)^25^ could also lead to the reduction in stroke associated with sufficient physical activity.

The WHO has specific recommendations for physical activity in which the term “more is better” is used. Recommendations for adults and older adults, as well as those living with chronic conditions, include 150–300 min per week of moderate‐to‐vigorous intensity physical activity, muscle‐strengthening activities at least twice per week, and limiting time spent sedentary (particularly screen time).^26^ Exercise is a potential prevention methodology for obesity for nearly all willing individuals; as observed by the CDC, primary prevention of overweight/obesity also reduces the risk for many other comorbidities, including but not limited to stroke.^27^ The AHA also recommends regular aerobic exercise as a role in stroke prevention and treatment.^28^ Our study found that stroke patients had a significantly higher prevalence of comorbidities, including overweight, congestive heart failure, coronary heart disease, angina, and heart attack, confirming the connection posited as part of CDC, WHO, and AHA guidelines. Our study also had the novel finding that those who did a high number of muscle strength exercises per week (>20 h per week) had a higher prevalence of stroke. This could be due to the high cortisol levels that the body releases in a state of constant stress. Notably, mortality rates have been correlated to increased serum cortisol levels in acute stroke patients.^29^ Thus, cortisol levels may play a role in causing stroke in patients doing muscle strength exercises >20 h per week and may deteriorate their outcomes. Therefore, the “more is better” recommendation should be revised to warn against strength exercises over 20 h per week.

Our findings also indicated that those who carried heavy loads due to their work had reduced stroke occurrence. This is corroborated by a large meta‐analysis that found that occupations with increased amounts of physical activity were associated with a 43% stroke risk reduction compared to sedentary workers and a 23% stroke risk reduction compared to those who only had moderate work‐based physical activity.^30^ The individuals with moderate physical activity at work had a decreased stroke risk of 36% compared to the sedentary workgroup.^30^

Since physical inactivity causes deteriorations in multiple mechanisms that cause stroke, it has been estimated that it could be the primary prevention of 10%–30% of stroke, depending on the training volume.^31^ Therefore, the choice of leisure time activities on health outcomes has significant clinical impacts. A study found that a high amount of inactivity during leisure time was associated with an increased stroke risk of 20%–25% compared to those who had high levels of activity in their leisure time, and a 15% increased risk when compared to those who did a moderate physical activity in their leisure time.^30^ In the current study, participants who stated they walked, bicycled, or did house or yard work over the past 30 days had a significant reduction in stroke prevalence, with increased durations having increased benefit.

Based on our results, sedentary behaviors such as watching TV, playing video games, or using a computer for more than 4 h per day were associated with a higher risk of stroke than those who did not engage in these behaviors. Sedentary behavior has been linked to obesity, cardiovascular, and cerebrovascular disease, even in young people.^32^, ^33^, ^34^, ^35^, ^36^ Moreover, research has demonstrated an association between excessive durations of sedentary behavior and poor cardiopulmonary function and reduced muscle mass, which would increase the risk of cardiovascular and cerebrovascular conditions.^37^, ^38^ In a study of 13,710 Chinese participants, sedentary behavior was a significant predictor for myocardial infarction and stroke.^33^ On further categorization of sedentary behavior durations, the study found that more extended time of sedentary behavior (≥8 h) had the highest risk compared to shorter durations (<4 h); however, no differences were found between short (<4 h) and middle (4–8 h) duration groups.^33^ The same findings were supported by Pandey et al.; who showed a nonlinear association between cardiovascular diseases and sedentary time with an increased risk at very high levels only.^39^ A study of 1993 Finnish individuals aged 30–45 found that TV viewing was the most consistently associated sedentary behavior with different adiposity markers (body mass index and waist circumference).^35^ The same study concluded that “obesogenic effects of TV viewing are partly mediated by other lifestyle factors,” which may explain our findings. Those who used the computer for up to 2 h had lower odds of stroke than those who did not use the computer at all. This may include other protective factors as being physically active or having a healthy diet, which would dilute the harmful effects of being sedentary.

Although the data utilized in this study did not include the COVID‐19 period, they may introduce the risks of COVID‐19‐related sedentary lifestyle. The current pandemic may play a role in increasing the incidence of stroke due to a reduction in physical activity and an increase in screen‐based sedentary behavior.^40^, ^41^, ^42^ One crucial oversight of the pandemic was that many countries' lockdown measures led to an exponential decline in daily step count across the globe.^43^ Some of the variables that lead to differences among regions were social distancing and home isolation enforcement levels. Even in places with low COVID‐19 counts that have not had to implement lockdowns (Japan, Taiwan, and South Korea), there has still been a decrease in the number of steps taken daily.^43^ This decrease in physical activity most certainly will have lasting adverse effects. This could be the beginning of an even deadlier pandemic with the long‐term effects of the virus on stroke and cardiovascular disease unknown.^44^

### Study limitations

The current findings have limitations; notably, our data come from a self‐report survey of cross‐sectional nature where a temporal relationship cannot be established. Although survey‐based data may be skewed by participants' recall bias, having a stroke is a momentous event that will be hard to miss or forget. Additionally, the stroke question was based on physicians informing the condition, not participants' perception or speculations. However, type of stroke was not reported in NHANES database. Furthermore, different causes of stroke were not assessed through NHANES database.

Errors in measuring physical activity might be another source of bias; however, by analyzing all questions measuring physical activity, the risk that measurement error impacted outcomes was mitigated. Participants' demographics and underlying conditions can affect the association's strength, which was reduced as much as possible using the propensity score matching methodology. Finally, sampling weights were not be incorporated into the matching procedure which may limit the generalizability to the original survey target population (whole US) but does not affect the validity of the results in reference to the included participants. That said, this study is the first nationally representative study, with a large sample size and long duration, to examine the association between physical activity and stroke odds.

## Conclusion

In a national cohort of the US population, a range of activity‐related behaviors was significantly associated with reduced stroke odds. The findings are debunking the “one‐size‐fits‐all” myth when it comes to behavioral changes; different types, frequencies, and intensities of physical activity showed association with reduced stroke incidence, implying that there is an option for everyone. Daily or every other day activities are more important in reducing the stroke risk than reducing sedentary behavior duration. Based on our findings, we call for a prospective study of activity‐related behavior participation and stroke incidence with implicating public health interventions of physical activity as a stroke prophylactic measure.

## Conflict of Interest

Kevin Kallmes works for and holds equity in Nested Knowledge, Superior Medical Experts, and Conway Medical.

## Supporting information


**Table S1.** Characteristics of the included participants.
**Table S2.** Description of the survey tool used for measuring physical activity.
**Table S3.** Aerobic Activities and stroke incidence.
**Table S4.** Aerobic Activities and stroke incidence – logistic regression results of the matched sample.
**Table S5.** Recreational activities and stroke incidence.
**Table S6.** Recreational activities and stroke incidence – logistic regression results of the matched sample.
**Table S7.** Muscle strengthening activities and stroke incidence.
**Table S8.** Muscle strengthening activities and stroke incidence – logistic regression results of the matched sample.
**Table S9.** Sedentary behavior and stroke incidence.
**Table S10.** Sedentary behavior and stroke incidence – logistic regression results of the matched sample.
**Table S11.** Differences in activity levels and stroke incidence.
**Table S12.** Differences in activity levels and stroke incidence – Logistic regression results of the matched sample.
