# Supplementary material for: Physical activity level and stroke risk in US population: A matched case–control study of 102,578 individuals
Source: Ann Clin Transl Neurol. 2022 Jan 30;9(3):264–75. doi: 10.1002/acn3.51511 (PMC8935290; doi:10.1002/acn3.51511)
Supplement: Supplementary file 1 — Table S1. Characteristics of the included participants. Table S2. Description of the survey tool used for measuring physical activity. Table S3. Aerobic Activities and stroke incidence. Table S4. Aerobic Activities and stroke incidence – logistic regression results of the matched sample. Table S5. Recreational activities and stroke incidence. Table S6. Recreational activities and stroke incidence – logistic regression results of the matched sample. Table S7. Muscle strengthening activities and stroke incidence. Table S8. Muscle strengthening activities and stroke incidence – logistic regression results of the matched sample. Table S9. Sedentary behavior and stroke incidence. Table S10. Sedentary behavior and stroke incidence – logistic regression results of the matched sample. Table S11. Differences in activity levels and stroke incidence. Table S12. Differences in activity levels and stroke incidence – Logistic regression results of the matched sample. [file ACN3-9-264-s001.docx]

**Supplementary Table 1.**  **Characteristics of the included participants**

| **Variables - n (%)** | **1999-2006** | | | | | | | **2007-2018** | | | | | | | | **2011-2016** | | | | | | |
| --- | --- | --- | --- | --- | --- | --- | --- | --- | --- | --- | --- | --- | --- | --- | --- | --- | --- | --- | --- | --- | --- | --- |
|  | **Overall** | **Stroke status (unmatched)** | | | **Stroke status (matched)** | | | **Overall** | **Stroke status (unmatched)** | | | | **Stroke status (matched)** | | | **Overall** | **Stroke status (unmatched)** | | | **Stroke status (matched)** | | |
|  |  | **No** | **Yes** | **P-value** | **No** | **Yes** | **P-value** |  | **No** | **Yes** | **P-value** | **No** | | **Yes** | **P-value** |  | **No** | **Yes** | **P-value** | **No** | **Yes** | **P-value** |
|  | **45178** | **43508** | **1670** |  | **1670** | **1670** |  | **40508** | **38939** | **1569** |  | **1569** | | **1569** |  | **16892** | **16280** | **612** |  | **612** | **612** |  |
| **Age (%)** | | | | | | | | | | | | | | | | | | | | | | |
| **20-29** | 8941 (19.8) | 8916 (20.5) | 25 ( 1.5) | <0.001* | 24 ( 1.4) | 25 ( 1.5) | 0.669 | 6718 (16.6) | 6696 (17.2) | 22 ( 1.4) | <0.001* | 18 ( 1.1) | | 22 ( 1.4) | 0.363 | 2921 (17.3) | 2911 (17.9) | 10 ( 1.6) | <0.001* | 9 ( 1.5) | 10 ( 1.6) | 0.919 |
| **30-39** | 7726 (17.1) | 7689 (17.7) | 37 ( 2.2) |  | 36 ( 2.2) | 37 ( 2.2) |  | 6858 (16.9) | 6814 (17.5) | 44 ( 2.8) |  | 38 ( 2.4) | | 44 ( 2.8) |  | 2928 (17.3) | 2914 (17.9) | 14 ( 2.3) |  | 14 ( 2.3) | 14 ( 2.3) |  |
| **40-49** | 7649 (16.9) | 7530 (17.3) | 119 ( 7.1) |  | 107 ( 6.4) | 119 ( 7.1) |  | 6797 (16.8) | 6678 (17.1) | 119 ( 7.6) |  | 96 ( 6.1) | | 119 ( 7.6) |  | 2866 (17.0) | 2824 (17.3) | 42 ( 6.9) |  | 37 ( 6.0) | 42 ( 6.9) |  |
| **50-59** | 5722 (12.7) | 5576 (12.8) | 146 ( 8.7) |  | 143 ( 8.6) | 146 ( 8.7) |  | 6465 (16.0) | 6236 (16.0) | 229 ( 14.6) |  | 221 (14.1) | | 229 ( 14.6) |  | 2758 (16.3) | 2654 (16.3) | 104 ( 17.0) |  | 95 (15.5) | 104 ( 17.0) |  |
| **60-69** | 6378 (14.1) | 6004 (13.8) | 374 ( 22.4) |  | 343 (20.5) | 374 ( 22.4) |  | 6728 (16.6) | 6312 (16.2) | 416 ( 26.5) |  | 404 (25.7) | | 416 ( 26.5) |  | 2765 (16.4) | 2603 (16.0) | 162 ( 26.5) |  | 158 (25.8) | 162 ( 26.5) |  |
| **70+** | 8762 (19.4) | 7793 (17.9) | 969 ( 58.0) |  | 1017 (60.9) | 969 ( 58.0) |  | 6942 (17.1) | 6203 (15.9) | 739 ( 47.1) |  | 792 (50.5) | | 739 ( 47.1) |  | 2654 (15.7) | 2374 (14.6) | 280 ( 45.8) |  | 299 (48.9) | 280 ( 45.8) |  |
| **Gender - Male (%)** | 21354 (47.3) | 20506 (47.1) | 848 ( 50.8) | 0.004* | 850 (50.9) | 848 ( 50.8) | 0.972 | 19606 (48.4) | 18841 (48.4) | 765 ( 48.8) | 0.793 | 793 (50.5) | | 765 ( 48.8) | 0.335 | 8157 (48.3) | 7865 (48.3) | 292 ( 47.7) | 0.803 | 305 (49.8) | 292 ( 47.7) | 0.493 |
| **Race (%)** | | | | | | | | | | | | | | | | | | | | | | |
| **Mexican American** | 9458 (20.9) | 9245 (21.2) | 213 ( 12.8) | <0.001* | 225 (13.5) | 213 ( 12.8) | 0.180 | 6236 (15.4) | 6095 (15.7) | 141 ( 9.0) | <0.001* | 139 ( 8.9) | | 141 ( 9.0) | 0.999 | 2276 (13.5) | 2214 (13.6) | 62 ( 10.1) | <0.001* | 68 (11.1) | 62 ( 10.1) | 0.883 |
| **Non-Hispanic Black** | 9101 (20.1) | 8730 (20.1) | 371 ( 22.2) |  | 367 (22.0) | 371 ( 22.2) |  | 8525 (21.0) | 8091 (20.8) | 434 ( 27.7) |  | 432 (27.5) | | 434 ( 27.7) |  | 3799 (22.5) | 3627 (22.3) | 172 ( 28.1) |  | 161 (26.3) | 172 ( 28.1) |  |
| **Non-Hispanic White** | 23134 (51.2) | 22134 (50.9) | 1000 ( 59.9) |  | 1017 (60.9) | 1000 ( 59.9) |  | 16851 (41.6) | 16078 (41.3) | 773 ( 49.3) |  | 776 (49.5) | | 773 ( 49.3) |  | 6317 (37.4) | 6042 (37.1) | 275 ( 44.9) |  | 275 (44.9) | 275 ( 44.9) |  |
| **Other Hispanic** | 1805 ( 4.0) | 1771 ( 4.1) | 34 ( 2.0) |  | 18 ( 1.1) | 34 ( 2.0) |  | 4244 (10.5) | 4142 (10.6) | 102 ( 6.5) |  | 105 ( 6.7) | | 102 ( 6.5) |  | 1832 (10.8) | 1786 (11.0) | 46 ( 7.5) |  | 53 ( 8.7) | 46 ( 7.5) |  |
| **Other Race - Including Multi-Racial** | 1680 ( 3.7) | 1628 ( 3.7) | 52 ( 3.1) |  | 43 ( 2.6) | 52 ( 3.1) |  | 4652 (11.5) | 4533 (11.6) | 119 ( 7.6) |  | 117 ( 7.5) | | 119 ( 7.6) |  | 2668 (15.8) | 2611 (16.0) | 57 ( 9.3) |  | 55 ( 9.0) | 57 ( 9.3) |  |
| **Education (%)** | | | | | | | | | | | | | | | | | | | | | | |
| **12th grade without diploma or less** | 13750 (30.4) | 13010 (29.9) | 740 ( 44.3) | <0.001* | 726 (43.5) | 740 ( 44.3) | 0.507 | 10298 (25.4) | 9745 (25.0) | 553 ( 35.2) | <0.001* | 562 (35.8) | | 553 ( 35.2) | 0.644 | 3892 (23.0) | 3683 (22.6) | 209 ( 34.2) | <0.001* | 221 (36.1) | 209 ( 34.2) | 0.583 |
| **College graduate or higher** | 8788 (19.5) | 8616 (19.8) | 172 ( 10.3) |  | 165 ( 9.9) | 172 ( 10.3) |  | 9171 (22.6) | 8968 (23.0) | 203 ( 12.9) |  | 223 (14.2) | | 203 ( 12.9) |  | 4244 (25.1) | 4160 (25.6) | 84 ( 13.7) |  | 73 (11.9) | 84 ( 13.7) |  |
| **High school graduate GED or equivalent** | 10684 (23.6) | 10310 (23.7) | 374 ( 22.4) |  | 358 (21.4) | 374 ( 22.4) |  | 9243 (22.8) | 8822 (22.7) | 421 ( 26.8) |  | 413 (26.3) | | 421 ( 26.8) |  | 3663 (21.7) | 3498 (21.5) | 165 ( 27.0) |  | 176 (28.8) | 165 ( 27.0) |  |
| **Some college courses or AA degree** | 11956 (26.5) | 11572 (26.6) | 384 ( 23.0) |  | 421 (25.2) | 384 ( 23.0) |  | 11796 (29.1) | 11404 (29.3) | 392 ( 25.0) |  | 371 (23.6) | | 392 ( 25.0) |  | 5093 (30.2) | 4939 (30.3) | 154 ( 25.2) |  | 142 (23.2) | 154 ( 25.2) |  |
| **Marital Status (%)** | | | | | | | | | | | | | | | | | | | | | | |
| **Divorced** | 4002 ( 8.9) | 3828 ( 8.8) | 174 ( 10.4) | <0.001* | 171 (10.2) | 174 ( 10.4) | 0.530 | 4432 (10.9) | 4199 (10.8) | 233 ( 14.9) | <0.001* | 226 (14.4) | | 233 ( 14.9) | 0.979 | 1818 (10.8) | 1726 (10.6) | 92 ( 15.0) | <0.001* | 90 (14.7) | 92 ( 15.0) | 0.978 |
| **Living with partner** | 2891 ( 6.4) | 2840 ( 6.5) | 51 ( 3.1) |  | 48 ( 2.9) | 51 ( 3.1) |  | 3276 ( 8.1) | 3213 ( 8.3) | 63 ( 4.0) |  | 71 ( 4.5) | | 63 ( 4.0) |  | 1411 ( 8.4) | 1387 ( 8.5) | 24 ( 3.9) |  | 29 ( 4.7) | 24 ( 3.9) |  |
| **Married** | 25036 (55.4) | 24230 (55.7) | 806 ( 48.3) |  | 768 (46.0) | 806 ( 48.3) |  | 20578 (50.8) | 19847 (51.0) | 731 ( 46.6) |  | 724 (46.1) | | 731 ( 46.6) |  | 8479 (50.2) | 8215 (50.5) | 264 ( 43.1) |  | 263 (43.0) | 264 ( 43.1) |  |
| **Never married** | 7133 (15.8) | 7040 (16.2) | 93 ( 5.6) |  | 92 ( 5.5) | 93 ( 5.6) |  | 7483 (18.5) | 7343 (18.9) | 140 ( 8.9) |  | 143 ( 9.1) | | 140 ( 8.9) |  | 3328 (19.7) | 3268 (20.1) | 60 ( 9.8) |  | 55 ( 9.0) | 60 ( 9.8) |  |
| **Separated** | 1421 ( 3.1) | 1367 ( 3.1) | 54 ( 3.2) |  | 47 ( 2.8) | 54 ( 3.2) |  | 1378 ( 3.4) | 1316 ( 3.4) | 62 ( 4.0) |  | 66 ( 4.2) | | 62 ( 4.0) |  | 570 ( 3.4) | 544 ( 3.3) | 26 ( 4.2) |  | 25 ( 4.1) | 26 ( 4.2) |  |
| **Widowed** | 4695 (10.4) | 4203 ( 9.7) | 492 ( 29.5) |  | 544 (32.6) | 492 ( 29.5) |  | 3361 ( 8.3) | 3021 ( 7.8) | 340 ( 21.7) |  | 339 (21.6) | | 340 ( 21.7) |  | 1286 ( 7.6) | 1140 ( 7.0) | 146 ( 23.9) |  | 150 (24.5) | 146 ( 23.9) |  |
| **CHF** | 1499 ( 3.3) | 1189 ( 2.7) | 310 ( 18.6) | <0.001* | 259 (15.5) | 310 ( 18.6) | 0.021 | 1286 ( 3.2) | 1014 ( 2.6) | 272 ( 17.3) | <0.001* | 241 (15.4) | | 272 ( 17.3) | 0.148 | 559 ( 3.3) | 446 ( 2.7) | 113 ( 18.5) | <0.001* | 92 (15.0) | 113 ( 18.5) | 0.126 |
| **CHD** | 1892 ( 4.2) | 1608 ( 3.7) | 284 ( 17.0) | <0.001* | 279 (16.7) | 284 ( 17.0) | 0.853 | 1638 ( 4.0) | 1357 ( 3.5) | 281 ( 17.9) | <0.001* | 259 (16.5) | | 281 ( 17.9) | 0.321 | 651 ( 3.9) | 537 ( 3.3) | 114 ( 18.6) | <0.001* | 108 (17.6) | 114 ( 18.6) | 0.711 |
| **Angina** | 1538 ( 3.4) | 1276 ( 2.9) | 262 ( 15.7) | <0.001* | 238 (14.3) | 262 ( 15.7) | 0.265 | 988 ( 2.4) | 804 ( 2.1) | 184 ( 11.7) | <0.001* | 155 ( 9.9) | | 184 ( 11.7) | 0.107 | 389 ( 2.3) | 322 ( 2.0) | 67 ( 10.9) | <0.001* | 61 (10.0) | 67 ( 10.9) | 0.640 |
| **Heart attack** | 1946 ( 4.3) | 1594 ( 3.7) | 352 ( 21.1) | <0.001* | 347 (20.8) | 352 ( 21.1) | 0.865 | 1660 ( 4.1) | 1338 ( 3.4) | 322 ( 20.5) | <0.001* | 290 (18.5) | | 322 ( 20.5) | 0.163 | 646 ( 3.8) | 530 ( 3.3) | 116 ( 19.0) | <0.001* | 113 (18.5) | 116 ( 19.0) | 0.883 |

GED: general Educational Development; AA: associate of arts; CHF: congestive heart failure; CHD: coronary heart disease; *Statistically significant at p<0.05

**Supplementary Table 2.** **Description of the survey tool used for measuring physical activity**

| **Variable** | **Description** | **Answers** |
| --- | --- | --- |
| **Walked or bicycled over past 30 days (%)¥** | The next series of questions are about physical activities that {you/SP} {have/has} done over the past 30 days. First I will ask about activities that are related to transportation. Then I'll ask about physical activities that {you/he/she} do at school or in {your/his/her} leisure time. Over the past 30 days, {have/has} {you/SP} walked or bicycled as part of getting to and from work, or school, or to do errands? | Yes, no, unable |
| **How long per day minutes (%)¥** | On those days when {you/SP} walked or bicycled, about how long did {you/s/he} spend altogether doing this (minutes)? | Range of values |
| **Tasks around home yard past 30 days (%)¥** | Over the past 30 days, did {you/SP} do any tasks in or around {your/his/her} home or yard for at least 10 minutes that required moderate or greater physical effort? By moderate physical effort I mean, tasks that caused light sweating or a slight to moder ate increase in {your/his/her} heart rate or breathing. [Such as raking leaves, mowing the lawn or heavy cleaning.] | Yes, no, unable |
| **Number of times past 30 days (%)¥** | [Over the past 30 days], how often did {you/SP} do these tasks in or around {your/his/her} home or yard, that is tasks requiring at least moderate effort? [Such as raking leaves, mowing the lawn or heavy cleaning.] PROBE: How many times per day, per week, or per month did {you/s/he} do these activities? | Range of values |
| **How long each time minutes (%)¥** | About how long did {you/SP} do these tasks each time (minutes)? | Range of values |
| **Vigorous activity over past 30 days (%)¥** | The next questions are about physical activities including exercise, sports, and physically active hobbies that {you/SP} may have done in {your/his/her} leisure time or at school over the past 30 days. First I will ask you about vigorous activities that cause heavy sweating or large increases in breathing or heart rate. Then I will ask you about moderate activities that cause only light sweating or a slight to moderate increase in breathing or heart rate. Over the past 30 days, did {you/SP} do any vigorous activities for at least 10 minutes that caused heavy sweating, or large increases in breathing or heart rate? Some examples are running, lap swimming, aerobics classes or fast bicycling. | Yes, no, unable |
| **Moderate activity over past 30 days (%)¥** | [Over the past 30 days], did {you/SP} do moderate activities for at least 10 minutes that cause only light sweating or a slight to moderate increase in breathing or heart rate? Some examples are brisk walking, bicycling for pleasure, golf, and dancing . | Yes, no, unable |
| **Average level of physical activity each day (%)**¥ | Please tell me which of these four sentences best describes {your/SP's} usual daily activities? [Daily activities may include {your/his/her} work, housework if {you are/s/he is} a homemaker, going to and attending classes if {you are/s/he is} a student, and what {you/s/he} normally {do/does} throughout a typical day if {you are/he/she is} a retiree or unemployed.] | sits during the day and does not walk very much. stands or walks about a lot during the day. lifts light load or has to climb stairs or hills often. does heavy work or carries heavy loads. |
| **Muscle strengthening activities (%)**¥ | Over the past 30 days, did {you/SP} do any physical activities specifically designed to strengthen {your/his/her} muscles such as lifting weights, push-ups or sit-ups? Include all such activities even if you have mentioned them before. | Yes, no, unable |
| **Number of times past 30 days (%)**¥ | [Over the past 30 days], how often did {you/SP} do these physical activities? [Activities designed to strengthen {your/his/her} muscles such as lifting weights, push-ups or sit-ups.] | Range of values |
| **Activity comparison last month last year (%)¥** | How does the amount of activity that you reported {for SP} for the past 30 days compare with {your/his/her} physical activity for the past 12 months? Over the past 30 days, {were you/was he/she} | Same, less, more |
| **Compare activity with others same age (%)¥** | (MEC Interview Version) Compared with most {boys/girls} {your/SP's} age, would you say that {you are/SP is}... (SP Interview Version) Compared with most {men/boys/women/girls} {your/SP's} age, would you say that {you are/s/he is} | Same, less, more |
| **Compare activity with 10 years ago (%)¥** | Compared with {yourself/himself/herself} 10 years ago, would you say that {you are/SP is} | Same, less, more |
| **Vigorous work activity = yes (%)+** | Next I am going to ask you about the time {you spend/SP spends} doing different types of physical activity in a typical week. Please answer these questions even if {you do not consider yourself/SP does not consider himself/herself} to be a physically active person. Think first about the time {you spend/SP spends} doing work. Think of work as the things that {you have/SP has} to do such as paid or unpaid work, studying or training, household chores, and yard work. In answering the following questions, 'vigorous-intensity activities' are activities that require hard physical effort and cause large increases in breathing or heart rate, and 'moderate-intensity activities' are activities that require moderate physical effort and cause small increases in breathing or heart rate. Does {your/SP's} work involve vigorous-intensity activity that causes large increases in breathing or heart rate like carrying or lifting heavy loads, digging or construction work for at least 10 minutes continuously? | Yes, no |
| **Days vigorous work (%)+** | PROBE IF NEEDED: Vigorous-intensity activity causes large increases in breathing or heart rate and is done for at least 10 minutes continuously. (SP interview version) HARD EDIT: 1-7. (MEC interview version) HARD EDIT: Less than 1 day or more than 7 days. Error Message: The number of days should be between 1 and 7. ENTER NUMBER OF DAYS | Range of values |
| **Minutes vigorous intensity work (%)+** | ROBE IF NEEDED: Think about a typical day when you do vigorous-intensity activities during your work. PROBE IF NEEDED: Vigorous-intensity activity causes large increases in breathing or heart rate and is done for at least 10 minutes continuously. (SP interview version) SOFT EDIT: >4 HOURS. SOFT EDIT WORDING: INTERVIEWER, YOU HAVE RECORDED THAT THE SP SPENDS MORE THAN 4 HOURS DOING VIGOROUS-INTENSITY ACTIVITIES AT WORK ON A TYPICAL DAY. PLEASE CONFIRM WITH SP THAT OVER 4 HOURS IS CORRECT. HARD EDIT: >24 HOURS. HARD EDIT: <10 MINUTES. ENTER NUMBER OF MINUTES OR HOURS (MEC interview version) SOFT EDIT: >4 hours. Error Message: INTERVIEWER, YOU HAVE RECORDED THAT THE SP SPENDS MORE THAN 4 HOURS DOING VIGOROUS-INTENSITY ACTIVITIES AT WORK ON A TYPICAL DAY. PLEASE CONFIRM WITH SP THAT OVER 4 HOURS IS CORRECT. HARD EDIT: Less than 10 minutes or 24 hours or more. Error Message: The time should be 10 minutes or more, but less than 24 hours. ENTER NUMBER (OF MINUTES OR HOURS) | Range of values |
| **Moderate work activity = yes (%)+** | Does {your/SP's} work involve moderate-intensity activity that causes small increases in breathing or heart rate such as brisk walking or carrying light loads for at least 10 minutes continuously? | Yes, no |
| **Number of days moderate work (%)+** | PROBE IF NEEDED: Moderate-intensity activity causes small increases in breathing or heart rate and is done for at least 10 minutes continuously. (SP interview version) HARD EDIT: 1-7. ENTER NUMBER OF DAYS. (MEC interview version) HARD EDIT: Less than 1 day or more than 7 days Error Message: The number of days should be between 1 and 7. ENTER NUMBER OF DAYS | Range of values |
| **Minutes moderate intensity work (%)+** | PROBE IF NEEDED: Think about a typical day when you do moderate-intensity activities during your work. PROBE IF NEEDED: Moderate-intensity activity causes small increases in breathing or heart rate and is done for at least 10 minutes continuously. (SP interview version) SOFT EDIT: >4 HOURS. SOFT EDIT WORDING: INTERVIEWER, YOU HAVE RECORDED THAT THE SP SPENDS MORE THAN 4 HOURS DOING MODERATE-INTENSITY ACTIVITIES AT WORK ON A TYPICAL DAY. PLEASE CONFIRM WITH SP THAT OVER 4 HOURS IS CORRECT. HARD EDIT: >24 HOURS. HARD EDIT: <10 MINUTES. ENTER NUMBER OF MINUTES OR HOURS (MEC interview version) SOFT EDIT: >4 hours. Error Message: INTERVIEWER, YOU HAVE RECORDED THAT THE SP SPENDS MORE THAN 4 HOURS DOING MODERATE-INTENSITY ACTIVITIES AT WORK ON A TYPICAL DAY. PLEASE CONFIRM WITH SP THAT OVER 4 HOURS IS CORRECT. HARD EDIT: Less than 10 minutes or 24 hours or more. Error Message: The time should be 10 minutes or more, but less than 24 hours. ENTER NUMBER (OF MINUTES OR HOURS) | Range of values |
| **Walk or bicycle = yes (%)+** | The next questions exclude the physical activity of work that you have already mentioned. Now I would like to ask you about the usual way {you travel/SP travels} to and from places. For example, to work, for shopping, to school. {Do you/Does SP} walk or use a bicycle for at least 10 minutes continuously to get to and from places? | Yes, no |
| **Number of days walk or bicycle (%)+** | (SP interview version) HARD EDIT: 1-7. ENTER NUMBER OF DAYS (MEC interview version) HARD EDIT: Less than 1 day or more than 7 days Error Message: The number of days should be between 1 and 7. ENTER NUMBER OF DAYS | Range of values |
| **Minutes walk bicycle for transportation (%)+** | PROBE IF NEEDED: Think about a typical day when you walk or bicycle for travel. (SP interview version) SOFT EDIT: >4 HOURS. SOFT EDIT WORDING: INTERVIEWER, YOU HAVE RECORDED THAT THE SP SPENDS MORE THAN 4 HOURS WALKING OR BICYCLING TO GET TO AND FROM PLACES ON A TYPICAL DAY. PLEASE CONFIRM WITH SP THAT OVER 4 HOURS IS CORRECT. HARD EDIT: >24 HOURS. HARD EDIT: <10 MINUTES. ENTER NUMBER OF MINUTES OR HOURS (MEC interview version) SOFT EDIT: >4 hours. Error Message: INTERVIEWER, YOU HAVE RECORDED THAT THE SP SPENDS MORE THAN 4 HOURS WALKING OR BICYCLING TO GET TO AND FROM PLACES ON A TYPICAL DAY. PLEASE CONFIRM WITH SP THAT OVER 4 HOURS IS CORRECT. HARD EDIT: Less than 10 minutes or 24 hours or more. Error Message: The time should be 10 minutes or more, but less than 24 hours. ENTER NUMBER (OF MINUTES OR HOURS) | Range of values |
| **Vigorous recreational activities = yes (%)+** | The next questions exclude the work and transportation activities that you have already mentioned. Now I would like to ask you about sports, fitness and recreational activities. {Do you/Does SP} do any vigorous-intensity sports, fitness, or recreational activities that cause large increases in breathing or heart rate like running or basketball for at least 10 minutes continuously? | Yes, no |
| **Days vigorous recreational activities (%)+** | PROBE IF NEEDED: Vigorous-intensity activity causes large increases in breathing or heart rate and is done for at least 10 minutes continuously. (SP interview version) HARD EDIT: 1-7. ENTER NUMBER OF DAYS (MEC interview version) HARD EDIT: Less than 1 day or more than 7 days Error Message: The number of days should be between 1 and 7. ENTER NUMBER OF DAYS | Range of values |
| **Minutes vigorous recreational activities (%)+** | PROBE IF NEEDED: Think about a typical day when you do vigorous-intensity sports, fitness or recreational activities. (SP interview version) SOFT EDIT: >4 HOURS. SOFT EDIT WORDING: INTERVIEWER, YOU HAVE RECORDED THAT THE SP SPENDS MORE THAN 4 HOURS DOING VIGOROUS-INTENSITY RECREATIONAL ACTIVITIES ON A TYPICAL DAY. PLEASE CONFIRM WITH SP THAT OVER 4 HOURS IS CORRECT. HARD EDIT: >24 HOURS. HARD EDIT: <10 MINUTES. ENTER NUMBER OF MINUTES OR HOURS (MEC interview version) SOFT EDIT: >4 hours. Error Message: INTERVIEWER, YOU HAVE RECORDED THAT THE SP SPENDS MORE THAN 4 HOURS DOING VIGOROUS-INTENSITY RECREATIONAL ACTIVITIES ON A TYPICAL DAY. PLEASE CONFIRM WITH SP THAT OVER 4 HOURS IS CORRECT. HARD EDIT: Less than 10 minutes or 24 hours or more. Error Message: The time should be 10 minutes or more, but less than 24 hours. ENTER NUMBER (OF MINUTES OR HOURS) | Range of values |
| **Moderate recreational activities = yes (%)+** | {Do you/Does SP} do any moderate-intensity sports, fitness, or recreational activities that cause a small increase in breathing or heart rate such as brisk walking, bicycling, swimming, or golf for at least 10 minutes continuously? | Yes, no |
| **Days moderate recreational activities (%)+** | PROBE IF NEEDED: Moderate-intensity sports, fitness or recreational activities cause small increases in breathing or heart rate and is done for at least 10 minutes continuously. (SP interview version) HARD EDIT: 1-7. ENTER NUMBER OF DAYS (MEC interview version) HARD EDIT: Less than 1 day or more than 7 days Error Message: The number of days should be between 1 and 7. ENTER NUMBER OF DAYS | Range of values |
| **Minutes moderate recreational activities (%)+** | PROBE IF NEEDED: Think about a typical day when you do moderate-intensity sports, fitness or recreational activities. PROBE IF NEEDED: Moderate-intensity sports, fitness or recreational activities cause small increases in breathing or heart rate and is done for at least 10 minutes continuously. (SP interview version) SOFT EDIT: >4 HOURS. SOFT EDIT WORDING: INTERVIEWER, YOU HAVE RECORDED THAT THE SP SPENDS MORE THAN 4 HOURS DOING MODERATE-INTENSITY RECREATIONAL ACTIVITIES ON A TYPICAL DAY. PLEASE CONFIRM WITH SP THAT OVER 4 HOURS IS CORRECT. HARD EDIT: >24 HOURS. HARD EDIT: <10 MINUTES. ENTER NUMBER OF MINUTES OR HOURS (MEC interview version) SOFT EDIT: >4 hours. Error Message: INTERVIEWER, YOU HAVE RECORDED THAT THE SP SPENDS MORE THAN 4 HOURS DOING MODERATE-INTENSITY RECREATIONAL ACTIVITIES ON A TYPICAL DAY. PLEASE CONFIRM WITH SP THAT OVER 4 HOURS IS CORRECT. HARD EDIT: Less than 10 minutes or 24 hours or more. Error Message; The time should be 10 minutes or more, but less than 24 hours. ENTER NUMBER (OF MINUTES OR HOURS) | Range of values |
| **Hours watch TV or videos past 30 days (%)#** | Now I will ask you first about TV watching and then about computer use. Over the past 30 days, on average how many hours per day did {you/SP} sit and watch TV or videos? Would you say . . . | Range of values |
| **Hours use computer past 30 days (%)#** | Over the past 30 days, on average how many hours per day did {you/SP} use a computer or play computer games outside of work or school? Include Playstation, Nintendo DS, or other portable video games. Would you say . . . | Range of values |

¥1999-2006 cohort; + 2007-2018 cohort; # 2011-2016 cohort

**Supplementary Table 3. Aerobic Activities and stroke incidence**

| **Variables - n (%)** | **Overall** | **Stroke status (unmatched)** | | | **Stroke status (matched)** | | |
| --- | --- | --- | --- | --- | --- | --- | --- |
|  |  | **No** | **Yes** | **P-value** | **No** | **Yes** | **P-value** |
|  |  |  |  |  |  |  |  |
| **Moderate activity over the past 30 days (%)¥** | | | | | | | |
| **No** | 22482 (49.8) | 21607 (49.7) | 875 ( 52.4) | <0.001* | 879 (52.6) | 875 ( 52.4) | <0.001* |
| **Yes** | 20987 (46.5) | 20500 (47.1) | 487 ( 29.2) |  | 625 (37.4) | 487 ( 29.2) |  |
| **Unable** | 1709 ( 3.8) | 1401 ( 3.2) | 308 ( 18.4) |  | 166 ( 9.9) | 308 ( 18.4) |  |
| **Walked or bicycled over the past 30 days (%)¥** | | | | | | | |
| **No** | 32929 (72.9) | 31791 (73.1) | 1138 ( 68.1) | <0.001* | 1173 (70.2) | 1138 ( 68.1) | <0.001* |
| **Yes** | 10678 (23.6) | 10424 (24.0) | 254 ( 15.2) |  | 345 (20.7) | 254 ( 15.2) |  |
| **Unable** | 1571 ( 3.5) | 1293 ( 3.0) | 278 ( 16.6) |  | 152 ( 9.1) | 278 ( 16.6) |  |
| **Walked or bicycled - How long per day (minutes) (%)¥** | | | | | | | |
| **1-20** | 24540 (54.3) | 23415 (53.8) | 1125 ( 67.4) | <0.001* | 939 (56.2) | 1125 ( 67.4) | <0.001* |
| **21-30** | 8361 (18.5) | 8061 (18.5) | 300 ( 18.0) |  | 285 (17.1) | 300 ( 18.0) |  |
| **31-60** | 9470 (21.0) | 9290 (21.4) | 180 ( 10.8) |  | 356 (21.3) | 180 ( 10.8) |  |
| **≥61** | 2807 ( 6.2) | 2742 ( 6.3) | 65 ( 3.9) |  | 90 ( 5.4) | 65 ( 3.9) |  |
| **Tasks around home yard over the past 30 days (%)¥** | | | | | | | |
| **No** | 18164 (40.2) | 17346 (39.9) | 818 ( 49.0) | <0.001* | 770 (46.1) | 818 ( 49.0) | <0.001* |
| **Yes** | 25530 (56.5) | 24937 (57.3) | 593 ( 35.5) |  | 758 (45.4) | 593 ( 35.5) |  |
| **Unable** | 1484 ( 3.3) | 1225 ( 2.8) | 259 ( 15.5) |  | 142 ( 8.5) | 259 ( 15.5) |  |
| **Tasks around home yard - Number of times over the past 30 days (%)¥** | | | | | | | |
| **1-3** | 12783 (28.3) | 12173 (28.0) | 610 ( 36.5) | <0.001* | 501 (30.0) | 610 ( 36.5) | <0.001* |
| **4** | 11934 (26.4) | 11514 (26.5) | 420 ( 25.1) |  | 434 (26.0) | 420 ( 25.1) |  |
| **5-13** | 9136 (20.2) | 8958 (20.6) | 178 ( 10.7) |  | 276 (16.5) | 178 ( 10.7) |  |
| **≥14** | 11325 (25.1) | 10863 (25.0) | 462 ( 27.7) |  | 459 (27.5) | 462 ( 27.7) |  |
| **Tasks around home yard - How long each time (minutes) (%)¥** | | | | | | | |
| **1-30** | 16750 (37.1) | 15813 (36.3) | 937 ( 56.1) | <0.001* | 730 (43.7) | 937 ( 56.1) | <0.001* |
| **31-60** | 15255 (33.8) | 14851 (34.1) | 404 ( 24.2) |  | 473 (28.3) | 404 ( 24.2) |  |
| **61-120** | 8396 (18.6) | 8225 (18.9) | 171 ( 10.2) |  | 280 (16.8) | 171 ( 10.2) |  |
| **≥121** | 4777 (10.6) | 4619 (10.6) | 158 ( 9.5) |  | 187 (11.2) | 158 ( 9.5) |  |
| **Moderate-intensity activities at work - for at least 10 minutes continuously (%)+** | | | | | | | |
| **Moderate work activity (%)** | 14382 (35.5) | 13993 (35.9) | 389 ( 24.8) | <0.001* | 482 (30.7) | 389 ( 24.8) | <0.001* |
| **Days of moderate-intensity activities at work - in a typical week (%)+** | | | | | | | |
| **1-2 days** | 15865 (39.2) | 14993 (38.5) | 872 ( 55.6) | <0.001* | 730 (46.5) | 872 ( 55.6) | <0.001* |
| **3-4 days** | 9039 (22.3) | 8682 (22.3) | 357 ( 22.8) |  | 274 (17.5) | 357 ( 22.8) |  |
| **5-6 days** | 8545 (21.1) | 8420 (21.6) | 125 ( 8.0) |  | 196 (12.5) | 125 ( 8.0) |  |
| **daily** | 7059 (17.4) | 6844 (17.6) | 215 ( 13.7) |  | 369 (23.5) | 215 ( 13.7) |  |
| **Minutes of moderate-intensity activities at work - on a typical day (%)+** | | | | | | | |
| **10-60 minutes** | 23856 (58.9) | 22795 (58.5) | 1061 ( 67.6) | <0.001* | 1056 (67.3) | 1061 ( 67.6) | 0.085 |
| **>60-120 minutes** | 5890 (14.5) | 5660 (14.5) | 230 ( 14.7) |  | 193 (12.3) | 230 ( 14.7) |  |
| **>120-240 minutes** | 5531 (13.7) | 5351 (13.7) | 180 ( 11.5) |  | 199 (12.7) | 180 ( 11.5) |  |
| **>240 minutes** | 5231 (12.9) | 5133 (13.2) | 98 ( 6.2) |  | 121 ( 7.7) | 98 ( 6.2) |  |
| **Walking or bicycling - for at least 10 minutes continuously (%)+** | | | | | | | |
| **Walking or bicycling (%)** | 10293 (25.4) | 10070 (25.9) | 223 ( 14.2) | <0.001* | 301 (19.2) | 223 ( 14.2) | <0.001* |
| **Days of walking or bicycling - in a typical week (%)+** | | | | | | | |
| **1-2 days** | 8322 (20.5) | 7924 (20.3) | 398 ( 25.4) | <0.001* | 260 (16.6) | 398 ( 25.4) | <0.001* |
| **3-4 days** | 10940 (27.0) | 10540 (27.1) | 400 ( 25.5) |  | 368 (23.5) | 400 ( 25.5) |  |
| **5-6 days** | 11206 (27.7) | 10866 (27.9) | 340 ( 21.7) |  | 431 (27.5) | 340 ( 21.7) |  |
| **daily** | 10040 (24.8) | 9609 (24.7) | 431 ( 27.5) |  | 510 (32.5) | 431 ( 27.5) |  |
| **Minutes of walking or bicycling for transportation - on a typical day (%)+** | | | | | | | |
| **10-60 minutes** | 35166 (86.8) | 33738 (86.6) | 1428 ( 91.0) | <0.001* | 1395 (88.9) | 1428 ( 91.0) | 0.131 |
| **>60-120 minutes** | 3652 ( 9.0) | 3552 ( 9.1) | 100 ( 6.4) |  | 122 ( 7.8) | 100 ( 6.4) |  |
| **>120-240 minutes** | 1083 ( 2.7) | 1051 ( 2.7) | 32 ( 2.0) |  | 34 ( 2.2) | 32 ( 2.0) |  |
| **>240 minutes** | 607 ( 1.5) | 598 ( 1.5) | 9 ( 0.6) |  | 18 ( 1.1) | 9 ( 0.6) |  |
| **Vigorous activity over the past 30 days (%)*** | | | | | | | |
| **No** | 29902 (66.2) | 28757 (66.1) | 1145 ( 68.6) | <0.001* | 1214 (72.7) | 1145 ( 68.6) | <0.001* |
| **Yes** | 12941 (28.6) | 12798 (29.4) | 143 ( 8.6) |  | 249 (14.9) | 143 ( 8.6) |  |
| **Unable** | 2335 ( 5.2) | 1953 ( 4.5) | 382 ( 22.9) |  | 207 (12.4) | 382 ( 22.9) |  |
| **Days of vigorous-intensity activities at work - in a typical week (%)+** | | | | | | | |
| **1-2 days** | 18356 (45.3) | 17355 (44.6) | 1001 ( 63.8) | <0.001* | 814 (51.9) | 1001 ( 63.8) | <0.001* |
| **3-4 days** | 8446 (20.9) | 8171 (21.0) | 275 ( 17.5) |  | 245 (15.6) | 275 ( 17.5) |  |
| **5-6 days** | 7707 (19.0) | 7593 (19.5) | 114 ( 7.3) |  | 167 (10.6) | 114 ( 7.3) |  |
| **daily** | 5999 (14.8) | 5820 (14.9) | 179 ( 11.4) |  | 343 (21.9) | 179 ( 11.4) |  |
| **Minutes vigorous-intensity activities at work - on a typical day (%)+** | | | | | | | |
| **10-60 minutes** | 24646 (60.8) | 23552 (60.5) | 1094 ( 69.7) | <0.001* | 1068 (68.1) | 1094 ( 69.7) | 0.060 |
| **>60-120 minutes** | 5277 (13.0) | 5081 (13.0) | 196 ( 12.5) |  | 170 (10.8) | 196 ( 12.5) |  |
| **>120-240 minutes** | 5275 (13.0) | 5098 (13.1) | 177 ( 11.3) |  | 198 (12.6) | 177 ( 11.3) |  |
| **>240 minutes** | 5310 (13.1) | 5208 (13.4) | 102 ( 6.5) |  | 133 ( 8.5) | 102 ( 6.5) |  |
| **Vigorous-intensity activities at work - for at least 10 minutes continuously (%)+** | | | | | | | |
| **Vigorous work activity (%)** | 7673 (18.9) | 7489 (19.2) | 184 ( 11.7) | <0.001* | 202 (12.9) | 184 ( 11.7) | 0.356 |

¥1999-2006 cohort; + 2007-2018 cohort; *Statistically significant

**Supplementary Table 4. Aerobic Activities and stroke incidence – Logistic regression results of the matched sample**

| **Variables** | **Logistic regression** | | | |
| --- | --- | --- | --- | --- |
|  | **OR** | **95% CI** | | **P-value** |
|  |  | **LL** | **UL** |  |
| **Moderate activity over the past 30 days¥** | | | | |
| No | Reference value | | | |
| Yes | 0.8 | 0.7 | 0.9 | 0.001* |
| Unable | 1.9 | 1.5 | 2.3 | <0.001* |
| **Walked or bicycled over the past 30 days¥** | | | | |
| No | Reference value | | | |
| Yes | 0.8 | 0.6 | 0.9 | 0.003* |
| Unable | 1.9 | 1.5 | 2.3 | <0.001* |
| **Walked or bicycled - How long per day (minutes) ¥** | | | | |
| 1-20 | Reference value | | | |
| 21-30 | 0.9 | 0.7 | 1.1 | 0.168 |
| 31-60 | 0.4 | 0.4 | 0.5 | <0.001* |
| ≥61 | 0.6 | 0.4 | 0.8 | 0.003* |
| **Tasks around home yard over the past 30 days¥** | | | | |
| No | Reference value | | | |
| Yes | 0.7 | 0.6 | 0.9 | <0.001* |
| Unable | 1.7 | 1.4 | 2.2 | <0.001* |
| **Tasks around home yard - Number of times over the past 30 days¥** | | | | |
| 1-3 | Reference value | | | |
| 4 | 0.8 | 0.7 | 1 | 0.012* |
| 5-13 | 0.5 | 0.4 | 0.7 | <0.001* |
| ≥14 | 0.8 | 0.7 | 1 | 0.033* |
| **Tasks around home yard - How long each time (minutes)¥** | | | | |
| 1-30 | Reference value | | | |
| 31-60 | 0.7 | 0.6 | 0.8 | <0.001* |
| 61-120 | 0.5 | 0.4 | 0.6 | <0.001* |
| ≥121 | 0.7 | 0.5 | 0.8 | <0.001* |
| **Moderate-intensity activities at work - for at least 10 minutes continuously+** | | | | |
| **Moderate work activity** | 0.7 | 0.6 | 0.9 | < 0.001* |
| **Days of moderate-intensity activities at work - in a typical week+** | | | | |
| 1-2 days | Reference value | | | |
| 3-4 days | 1.1 | 0.9 | 1.3 | 0.359 |
| 5-6 days | 0.5 | 0.4 | 0.7 | < 0.001* |
| daily | 0.5 | 0.4 | 0.6 | < 0.001* |
| **Minutes of moderate-intensity activities at work - on a typical day+** | | | | |
| 10-60 minutes | Reference value | | | |
| >60-120 minutes | 1.2 | 1 | 1.5 | 0.11 |
| >120-240 minutes | 0.9 | 0.7 | 1.1 | 0.347 |
| >240 minutes | 0.8 | 0.6 | 1.1 | 0.131 |
| **Walking or bicycling** **- for at least 10 minutes continuously+** | | | | |
| **Walking or bicycling** | 0.7 | 0.6 | 0.8 | < 0.001* |
| **Days of walking or bicycling - in a typical week+** | | | | |
| 1-2 days | Reference value | | | |
| 3-4 days | 0.7 | 0.6 | 0.9 | 0.001* |
| 5-6 days | 0.5 | 0.4 | 0.6 | < 0.001* |
| daily | 0.6 | 0.5 | 0.7 | < 0.001* |
| **Minutes of walking or bicycling for transportation** - **on a typical day+** | | | | |
| 10-60 minutes | Reference value | | | |
| >60-120 minutes | 0.8 | 0.6 | 1.1 | 0.113 |
| >120-240 minutes | 0.9 | 0.6 | 1.5 | 0.736 |
| >240 minutes | 0.5 | 0.2 | 1.1 | 0.08 |
| **Vigorous activity over the past 30 days¥** | | | | |
| No | Reference value | | | |
| Yes | 0.6 | 0.5 | 0.8 | <0.001* |
| Unable | 2 | 1.6 | 2.4 | <0.001* |
| **Days of vigorous-intensity activities at work - in a typical week+** | | | | |
| 1-2 days | Reference value | | | |
| 3-4 days | 0.9 | 0.8 | 1.1 | 0.36 |
| 5-6 days | 0.6 | 0.4 | 0.7 | < 0.001* |
| daily | 0.4 | 0.3 | 0.5 | < 0.001* |
| **Minutes vigorous-intensity activities at work - on a typical day+** | | | | |
| 10-60 minutes | Reference value | | | |
| >60-120 minutes | 1.1 | 0.9 | 1.4 | 0.297 |
| >120-240 minutes | 0.9 | 0.7 | 1.1 | 0.224 |
| >240 minutes | 0.7 | 0.6 | 1 | 0.037* |
| **Vigorous-intensity activities at work - for at least 10 minutes continuously+** | | | | |
| **Vigorous work activity** | 0.9 | 0.7 | 1.1 | 0.328 |

¥1999-2006 cohort; + 2007-2018 cohort; OR: odds ration; CI: confidence interval; LL: lower level; UL: upper level; *Statistically significant

**Supplementary Table 5. Recreational Activities and stroke incidence**

| **Variables - n (%)** | **Overall** | **Stroke status (unmatched)** | | | **Stroke status (matched)** | | |
| --- | --- | --- | --- | --- | --- | --- | --- |
|  |  | **No** | **Yes** | **P-value** | **No** | **Yes** | **P-value** |
|  |  |  |  |  |  |  |  |
| **Vigorous recreational activities - for at least 10 minutes continuously (%)+** | | | | | | | |
| **Vigorous recreational activities (%)** | 8567 (21.1) | 8491 (21.8) | 76 (4.8) | <0.001* | 153 ( 9.8) | 76 (4.8) | <0.001* |
| **Days of vigorous recreational activities - in a typical week (%)+** | | | | | | | |
| **1-2 days** | 12008 (29.6) | 11540 (29.6) | 468 (29.8) | <0.001* | 309 (19.7) | 468 (29.8) | <0.001* |
| **3-4 days** | 14555 (35.9) | 14057 (36.1) | 498 (31.7) |  | 453 (28.9) | 498 (31.7) |  |
| **5-6 days** | 8088 (20.0) | 7768 (19.9) | 320 (20.4) |  | 398 (25.4) | 320 (20.4) |  |
| **daily** | 5857 (14.5) | 5574 (14.3) | 283 (18.0) |  | 409 (26.1) | 283 (18.0) |  |
| **Minutes of vigorous recreational activities - on a typical day (%)+** | | | | | | | |
| **10-60 minutes** | 32540 (80.3) | 31132 (80.0) | 1408 ( 89.7) | <0.001* | 1356 (86.4) | 1408 (89.7) | 0.029* |
| **>60-120 minutes** | 5812 (14.3) | 5687 (14.6) | 125 (8.0) |  | 162 (10.3) | 125 (8.0) |  |
| **>120-240 minutes** | 2059 (5.1) | 2023 ( 5.2) | 36 (2.3) |  | 50 ( 3.2) | 36 (2.3) |  |
| **>240 minutes** | 97 (0.2) | 97 ( 0.2) | 0 (0.0) |  | 1 ( 0.1) | 0 (0.0) |  |
| **Moderate recreational activities - for at least 10 minutes continuously (%)+** | | | | | | | |
| **Moderate recreational activities (%)** | 15889 (39.2) | 15499 (39.8) | 390 (24.9) | <0.001* | 523 (33.3) | 390 (24.9) | <0.001* |
| **Days of moderate recreational activities - in a typical week (%)+** | | | | | | | |
| **1-2 days** | 12142 (30.0) | 11763 (30.2) | 379 (24.2) | <0.001* | 286 (18.2) | 379 (24.2) | <0.001* |
| **3-4 days** | 12941 (31.9) | 12473 (32.0) | 468 (29.8) |  | 400 (25.5) | 468 (29.8) |  |
| **5-6 days** | 7905 (19.5) | 7590 (19.5) | 315 (20.1) |  | 399 (25.4) | 315 (20.1) |  |
| **daily** | 7520 (18.6) | 7113 (18.3) | 407 (25.9) |  | 484 (30.8) | 407 (25.9) |  |
| **Minutes of moderate recreational activities - on a typical day (%)+** | | | | | | | |
| **10-60 minutes** | 32961 (81.4) | 31563 (81.1) | 1398 (89.1) | <0.001* | 1336 (85.1) | 1398 (89.1) | 0.003* |
| **>60-120 minutes** | 5014 (12.4) | 4895 (12.6) | 119 (7.6) |  | 150 (9.6) | 119 (7.6) |  |
| **>120-240 minutes** | 2249 (5.6) | 2209 (5.7) | 40 (2.5) |  | 71 (4.5) | 40 (2.5) |  |
| **>240 minutes** | 284 (0.7) | 272 (0.7) | 12 (0.8) |  | 12 (0.8) | 12 (0.8) |  |

+ 2007-2018 cohort; *Statistically significant

**Supplementary Table 6. Recreational Activities and stroke incidence – Logistic regression results of the matched sample**+

| **Variables** | **Logistic regression (matched)** | | | |
| --- | --- | --- | --- | --- |
|  | **OR** | **95% CI** | | **P-value** |
|  |  | **LL** | **UL** |  |
| **Vigorous recreational activities - for at least 10 minutes continuously** | | | | |
| **Vigorous recreational activities** | 0.5 | 0.4 | 0.6 | < 0.001* |
| **Days of vigorous recreational activities - in a typical week** | | | | |
| 1-2 days | Reference value | | | |
| 3-4 days | 0.7 | 0.6 | 0.9 | 0.001* |
| 5-6 days | 0.5 | 0.4 | 0.7 | < 0.001* |
| daily | 0.5 | 0.4 | 0.6 | < 0.001* |
| **Minutes of vigorous recreational activities - on a typical day** | | | | |
| 10-60 minutes | Reference value | | | |
| >60-120 minutes | 0.7 | 0.6 | 0.9 | 0.018* |
| >120-240 minutes | 0.7 | 0.4 | 1.1 | 0.099 |
| >240 minutes | NA | | | |
| **Moderate recreational activities - for at least 10 minutes continuously** | | | | |
| **Moderate recreational activities** | 0.7 | 0.6 | 0.8 | < 0.001* |
| **Days of moderate recreational activities - in a typical week** | | | | |
| 1-2 days | Reference value | | | |
| 3-4 days | 0.9 | 0.7 | 1.1 | 0.230 |
| 5-6 days | 0.6 | 0.5 | 0.7 | < 0.001* |
| daily | 0.6 | 0.5 | 0.8 | < 0.001* |
| **Minutes of moderate recreational activities - on a typical day** | | | | |
| 10-60 minutes | Reference value | | | |
| >60-120 minutes | 0.8 | 0.6 | 1 | 0.031* |
| >120-240 minutes | 0.5 | 0.4 | 0.8 | 0.002* |
| >240 minutes | 1 | 0.4 | 2.2 | 0.912 |

+ 2007-2018 cohort; OR: odds ration; CI: confidence interval; LL: lower level; UL: upper level; *Statistically significant

**Supplementary Table 7. Muscle strengthening activities and stroke incidence**

| **Variables - n (%)** | **Overall** | **Stroke status (unmatched)** | | | **Stroke status (matched)** | | |
| --- | --- | --- | --- | --- | --- | --- | --- |
|  |  | **No** | **Yes** | **P-value** | **No** | **Yes** | **P-value** |
|  |  |  |  |  |  |  |  |
| **Muscle strengthening activities (%)¥** | | | | | | | |
| **No** | 32474 (71.9) | 31287 (71.9) | 1187 (71.1) | <0.001* | 1245 (74.6) | 1187 (71.1) | <0.001* |
| **Yes** | 10994 (24.3) | 10827 (24.9) | 167 (10.0) |  | 275 (16.5) | 167 (10.0) |  |
| **Unable** | 1710 ( 3.8) | 1394 ( 3.2) | 316 (18.9) |  | 150 ( 9.0) | 316 (18.9) |  |
| **Muscle strengthening activities - Number of times past 30 days (%)¥** | | | | | | | |
| **1-7** | 11243 (24.9) | 11006 (25.3) | 237 (14.2) | <0.001* | 252 (15.1) | 237 (14.2) | 0.001* |
| **8-13** | 17120 (37.9) | 16614 (38.2) | 506 (30.3) |  | 555 (33.2) | 506 (30.3) |  |
| **14-20** | 2044 (4.5) | 2017 (4.6) | 27 (1.6) |  | 54 ( 3.2) | 27 (1.6) |  |
| **≥21** | 14771 (32.7) | 13871 (31.9) | 900 (53.9) |  | 809 (48.4) | 900 (53.9) |  |

¥1999-2006 cohort; *Statistically significant

**Supplementary Table 8. Muscle strengthening activities and stroke incidence – Logistic regression results of the matched sample¥**

| **Variables** | **Logistic regression (matched)** | | | |
| --- | --- | --- | --- | --- |
|  | **OR** | **95% CI** | | **P-value** |
|  |  | **LL** | **UL** |  |
| **Muscle strengthening activities** | | | | |
| No | Reference value | | | |
| Yes | 0.6 | 0.5 | 0.8 | <0.001* |
| Unable | 2.2 | 1.8 | 2.7 | <0.001* |
| **Muscle strengthening activities - Number of times past 30 days** | | | | |
| 1-7 | Reference value | | | |
| 8-13 | 1 | 0.8 | 1.2 | 0.776 |
| 14-20 | 0.5 | 0.3 | 0.9 | 0.012* |
| ≥21 | 1.2 | 1 | 1.4 | 0.102 |

¥1999-2006 cohort; OR: odds ration; CI: confidence interval; LL: lower level; UL: upper level; *Statistically significant

**Supplementary Table 9. Sedentary Behavior and stroke incidence**

| **Variables - n (%)** | **Overall** | **Stroke status (unmatched)** | | | **Stroke status (matched)** | | |
| --- | --- | --- | --- | --- | --- | --- | --- |
|  |  | **No** | **Yes** | **P-value** | **No** | **Yes** | **P-value** |
| **Minutes of sedentary activity - on a typical day (%)+** | | | | | | | |
| **0-60 minutes** | 2425 ( 6.0) | 2358 ( 6.1) | 67 ( 4.3) | <0.001* | 68 ( 4.3) | 67 ( 4.3) | <0.001* |
| **>60-120 minutes** | 4437 (11.0) | 4328 (11.1) | 109 ( 6.9) |  | 161 (10.3) | 109 ( 6.9) |  |
| **>120-240 minutes** | 10262 (25.3) | 9952 (25.6) | 310 ( 19.8) |  | 387 (24.7) | 310 ( 19.8) |  |
| **>240 minutes** | 23384 (57.7) | 22301 (57.3) | 1083 ( 69.0) |  | 953 (60.7) | 1083 ( 69.0) |  |
| **Daily hours of TV video or computer use (%)¥** | | | | | | | |
| **None** | 147 ( 0.3) | 146 ( 0.3) | 1 ( 0.1) | <0.001* | 7 ( 0.4) | 1 ( 0.1) | <0.001* |
| **< 2 hours** | 11267 (24.9) | 11016 (25.3) | 251 ( 15.0) |  | 358 (21.4) | 251 ( 15.0) |  |
| **2-4 hours** | 27519 (60.9) | 26775 (61.5) | 744 ( 44.6) |  | 902 (54.0) | 744 ( 44.6) |  |
| **> 4 hours** | 6245 (13.8) | 5571 (12.8) | 674 ( 40.4) |  | 403 (24.1) | 674 ( 40.4) |  |
| **TV or videos watching hours during the past 30 days (%)#** | | | | | | | |
| **None** | 516 ( 3.1) | 499 ( 3.1) | 17 ( 2.8) | <0.001* | 12 ( 2.0) | 17 ( 2.8) | <0.001* |
| **< 2 hours** | 4793 (28.4) | 4697 (28.9) | 96 ( 15.7) |  | 120 (19.6) | 96 ( 15.7) |  |
| **2-4 hours** | 8796 (52.1) | 8532 (52.4) | 264 ( 43.1) |  | 321 (52.5) | 264 ( 43.1) |  |
| **> 4 hours** | 2787 (16.5) | 2552 (15.7) | 235 ( 38.4) |  | 159 (26.0) | 235 ( 38.4) |  |
| **Computer using hours during the past 30 days (%)#** | | | | | | | |
| **None** | 5378 (31.8) | 5059 (31.1) | 319 ( 52.1) | <0.001* | 289 (47.2) | 319 ( 52.1) | 0.017* |
| **< 2 hours** | 6751 (40.0) | 6581 (40.4) | 170 ( 27.8) |  | 221 (36.1) | 170 ( 27.8) |  |
| **2-4 hours** | 3760 (22.3) | 3669 (22.5) | 91 ( 14.9) |  | 77 (12.6) | 91 ( 14.9) |  |
| **> 4 hours** | 1003 ( 5.9) | 971 ( 6.0) | 32 ( 5.2) |  | 25 ( 4.1) | 32 ( 5.2) |  |

+ 2007-2018 cohort; ¥1999-2006 cohort; # 2011-2016 cohort; *Statistically significant

**Supplementary Table 10. Sedentary Behavior and stroke incidence – Logistic regression results of the matched sample**

| **Variables** | **Logistic regression (matched)** | | | |
| --- | --- | --- | --- | --- |
|  | **OR** | **95% CI** | | **P-value** |
|  |  | **LL** | **UL** |  |
| **Minutes of sedentary activity - on a typical day+** | | | | |
| 0-60 minutes | Reference value | | | |
| >60-120 minutes | 0.7 | 0.5 | 1 | 0.077 |
| >120-240 minutes | 0.8 | 0.6 | 1.2 | 0.271 |
| >240 minutes | 1.2 | 0.8 | 1.6 | 0.422 |
| **Daily hours of TV video or computer use¥** | | | | |
| None- <2 hours | Reference value | | | |
| 2-4 hours | 1.19 | 0.99 | 1.44 | 0.060 |
| > 4 hours | 2.42 | 1.97 | 2.96 | <0.001* |
| **TV or videos watching hours during the past 30 days#** | | | | |
| None- <2 hours | Reference value | | | |
| 2-4 hours | 0.96 | 0.71 | 1.29 | 0.790 |
| > 4 hours | 1.72 | 1.25 | 2.38 | <0.001* |
| **Computer using hours during the past 30 days#** | | | | |
| None- <2 hours | Reference value | | | |
| 2-4 hours | 1.23 | 0.88 | 1.71 | 0.210 |
| > 4 hours | 1.33 | 0.78 | 2.3 | 0.290 |
| **Activity for the past 30 days compared with physical activity for the past 12 months¥** | | | | |
| Same | Reference value | | | |
| Less | 1.3 | 1.1 | 1.5 | 0.002* |
| More | 1.1 | 0.9 | 1.4 | 0.421 |

¥1999-2006 cohort; + 2007-2018 cohort; # 2011-2016 cohort; OR: odds ration; CI: confidence interval; LL: lower level; UL: upper level; *Statistically significant

**Supplementary Table 11. Differences in activity levels and stroke incidence¥**

| **Variables - n (%)** | **Overall** | **Stroke status (unmatched)** | | | | **Stroke status (matched)** | | |
| --- | --- | --- | --- | --- | --- | --- | --- | --- |
|  |  | **No** | **Yes** | **P-value** | **No** | | **Yes** | **P-value** |
| **Average level of physical activity each day (%)** | | | | | | | | |
| **Sits during the day and does not walk very much** | 23344 (51.7) | 10712 (24.6) | 828 (49.6) | <0.001* | 644 (38.6) | | 828 (49.6) | <0.001* |
| **Stands or walks about a lot during the day** | 11540 (25.5) | 22670 (52.1) | 674 (40.4) |  | 801 (48.0) | | 674 (40.4) |  |
| **Lifts light load or has to climb stairs or hills often** | 7177 (15.9) | 7025 (16.1) | 152 (9.1) |  | 172 (10.3) | | 152 (9.1) |  |
| **Does heavy work or carries heavy loads** | 3117 ( 6.9) | 3101 ( 7.1) | 16 (1.0) |  | 53 ( 3.2) | | 16 (1.0) |  |
| **Activities during the last month compared to the preceding 12 months (%)** | | | | | | | | |
| **Same** | 27547 (61.0) | 26533 (61.0) | 1014 (60.7) | <0.001* | 1092 (65.4) | | 1014 ( 60.7) | 0.009* |
| **Less** | 10143 (22.5) | 9677 (22.2) | 466 (27.9) |  | 391 (23.4) | | 466 ( 27.9) |  |
| **More** | 7488 (16.6) | 7298 (16.8) | 190 (11.4) |  | 187 (11.2) | | 190 ( 11.4) |  |
| **Compare activity with others at the same age (%)** | | | | | | | | |
| **Same** | 19223 (42.5) | 18689 (43.0) | 534 (32.0) | <0.001* | 643 (38.5) | | 534 (32.0) | <0.001* |
| **Less** | 9321 (20.6) | 8730 (20.1) | 591 (35.4) |  | 329 (19.7) | | 591 (35.4) |  |
| **More** | 16634 (36.8) | 16089 (37.0) | 545 (32.6) |  | 698 (41.8) | | 545 (32.6) |  |
| **Compare activity with 10 years ago (%)** | | | | | | | | |
| **Same** | 10450 (23.1) | 10226 (23.5) | 224 (13.4) | <0.001* | 362 (21.7) | | 224 (13.4) | <0.001* |
| **Less** | 30063 (66.5) | 28738 (66.1) | 1325 (79.3) |  | 1195 (71.6) | | 1325 (79.3) |  |
| **More** | 4665 (10.3) | 4544 (10.4) | 121 (7.2) |  | 113 ( 6.8) | | 121 (7.2) |  |

¥1999-2006 cohort; *Statistically significant

**Supplementary Table 12. Differences in activity levels and stroke incidence – Logistic regression results of the matched sample¥**

| **Variables** | **Logistic regression (matched)** | | | |
| --- | --- | --- | --- | --- |
|  | **OR** | **95% CI** | | **P-value** |
|  |  | **LL** | **UL** |  |
| **Average level of physical activity each day** | | | | |
| Sits during the day and does not walk very much | Reference value | | | |
| Stands or walks about a lot during the day | 0.7 | 0.6 | 0.8 | <0.001* |
| Lifts light load or has to climb stairs or hills often | 0.7 | 0.5 | 0.9 | 0.002* |
| Does heavy work or carries heavy loads | 0.2 | 0.1 | 0.4 | <0.001* |
| **Activities during the last month compared to the preceding 12 months (%)** | | | | |
| Same | Reference value | | | |
| Less | 1.3 | 1.1 | 1.5 | 0.002* |
| More | 1.1 | 0.9 | 1.4 | 0.421 |
| **Compare activity with others at the same age** | | | | |
| Same | Reference value | | | |
| Less | 2.2 | 1.8 | 2.6 | <0.001* |
| More | 0.9 | 0.8 | 1.1 | 0.451 |
| **Compare activity with 10 years ago** | | | | |
| Same | Reference value | | | |
| Less | 1.8 | 1.5 | 2.2 | <0.001* |
| More | 1.7 | 1.3 | 2.4 | <0.001* |

¥1999-2006 cohort; OR: odds ration; CI: confidence interval; LL: lower level; UL: upper level; *Statistically significant
